# Experimental Validation of Large Eddy Simulation as a Benchmark for Reynolds-Averaged Navier-Stokes Flow Modeling in a Magnetically Levitated Blood Pump

**DOI:** 10.1007/s10439-025-03846-4

**Published:** 2025-12-09

**Authors:** Jonas Abeken, Utku Gülan, Kai von Petersdorff-Campen, Vasileios Charitatos, Marianne Schmid Daners, Mirko Meboldt, Markus Holzner, Diane de Zélicourt, Vartan Kurtcuoglu

**Affiliations:** 1 Interface Group, Department of Physiology, University of Zurich, Zurich, Switzerland; 2 Hi-D Imaging, Winterthur, Switzerland; 3 Product Development Group Zurich, Institute of Materials, Design and Fabrication, ETH Zurich, Zurich, Switzerland; 4 Institute for Dynamic Systems and Control, ETH Zurich, Zurich, Switzerland; 5 Environmental Fluid Mechanics Group, Institute of Hydraulic Engineering and River Research, University of Natural Resources and Life Sciences BOKU, Vienna, Austria

**Keywords:** Ventricular assist device, Computational fluid dynamics, LES, RANS, Velocimetry, PIV, MagLev

## Abstract

**Purpose:**

This study investigates the potential of large eddy simulation (LES) as a benchmark for validating Reynolds-averaged Navier-Stokes (RANS) models in the CentriMag centrifugal blood pump. We compare velocity field predictions from LES and RANS against particle image velocimetry (PIV) and quantify previously unreported non-rotatory components in the motion of the magnetically levitated impeller, assessing their impact on flow dynamics.

**Methods:**

We performed PIV on an optically accessible pump replica and compared phase-averaged velocity fields with computational fluid dynamics (CFD) predictions from LES and three unsteady RANS models. In addition, using custom optical trackers embedded in the PIV setup, we quantified the three-dimensional impeller motion and replicated its main non-rotatory components in the simulations.

**Results:**

The impeller exhibited complex but small deviations from ideal rotation. When incorporated into the CFD model, these had negligible impact on the flow field. LES predictions agreed well with PIV data, with root-mean-square velocity errors of approximately 3%, while the RANS models showed larger local deviations, particularly in the outlet region.

**Conclusion:**

LES demonstrated close agreement with PIV and proved to be a reliable reference for validating RANS models in this study. Although the analysis was limited to a single pump, LES warrants further consideration as a potential benchmark method that could reduce reliance on extensive experimental flow validation. Consistent with previous findings, the transition from the volute to the outlet emerged as a particularly sensitive region for RANS modeling and validation. The quantified non-rotatory impeller motion had negligible impact on the flow field, supporting the continued use of idealized rotor motion in CFD modeling of similar magnetically levitated devices.

**Supplementary Information:**

The online version contains supplementary material available at 10.1007/s10439-025-03846-4.

## Introduction

The development of ventricular assist devices (VADs) is a complex, multi-disciplinary endeavor, where biological challenges such as thrombogenesis and hemolysis must be addressed, and electromechanical targets such as overall size and reliability need to be met. Fluid dynamics are a critical aspect at the intersection of the two requirement areas: the pump’s power consumption is directly influenced by its hydrodynamic efficiency, while flow-induced stresses have a substantial effect on platelet activation and blood damage. Consequently, analyzing and optimizing the flow path is a focus in VAD development. This is approached experimentally using in vitro models and numerically through computational fluid dynamics (CFD). In vitro models allow for rapid measurements across various operating conditions and pump geometries, whereas CFD provides unrivaled detail in pressure and velocity fields across the entire fluid domain. This facilitates the derivation of forces acting on the rotor and fluid stresses acting on blood components [1–5].

Recognizing the importance of CFD in the development of medical devices, the United States Food and Drug Administration (FDA) has prioritized computational modeling in its regulatory science initiatives [6]. To assess the reliability and accuracy of CFD in blood-contacting medical devices, the FDA-sponsored interlaboratory studies for blood flow simulations in two benchmark geometries [7–12]. These efforts contributed to the development of the ASME V&V 40-2018 standard [13] and an FDA guidance document [14], advancing the standardization and, with it, the reliability of CFD investigations in medical device development, including those involving VADs. However, the FDA initiatives also highlighted the variability in CFD prediction accuracy depending on specific model choices and simulation parameters.

One of the two FDA benchmark geometries was a generic blood pump, for which the FDA collected computational predictions of pressure and velocity fields as well as hemolysis levels from 24 independent laboratories and compared them to experimental measurements from three laboratories. In the final evaluation of this inter-laboratory study, Ponnaluri et al. concluded that “no single participant was able to accurately predict all quantities of interest (pressure head, velocity, and hemolysis) at all conditions” [9]. Accurate prediction was classified as a mean relative error below 20%. Each participant reported values with a mean error > 20% for at least one predicted quantity in at least one condition. The least accurate predictions occurred for the pump outlet, an area characterized by a prominent jet and flow separation. All CFD entries employed either the steady or unsteady Reynolds-Averaged Navier-Stokes (RANS) approach. RANS models are attractive due to their computational efficiency, but their accuracy can be sensitive to the choice of the turbulence closure model, especially in regions of separation like the FDA pump’s outlet diffuser. While several studies have demonstrated good agreement between RANS-based simulations and experimental data in blood pumps, the findings by Ponnaluri et al. underscore the importance of rigorous validation of CFD models. Although their analysis considered different choices of turbulence models and grid structures, the study’s open design and consequent variability in the model setup limited the ability to draw definitive conclusions regarding the impact of individual modeling choices on simulation accuracy.

Notably, Ponnaluri et al. did not evaluate the variability of the experimental reference data itself. This issue was addressed in an earlier study by Hariharan et al. [8], who analyzed the reproducibility of particle image velocimetry (PIV) measurements across three laboratories using the same benchmark pump. They quantified the inter-laboratory variability using the coefficient of variation (CoV), defined as the ratio of the standard deviation between laboratories to the mean. Depending on the operating condition, the CoV reached up to 33% for the measured pressure head. For velocity fields, the average CoV ranged between 7% and 11% across a section of the volute and reached up to 35% in the outlet diffuser region [8]. It is therefore evident that experimental measurements are not the absolute reference they are often perceived to be.

The accuracy of both experimental measurements and CFD simulations is influenced by multiple factors. PIV measurements, as used in the FDA-sponsored study, can be affected by optical reflection and refraction or a low signal-to-noise ratio. CFD results are sensitive to the underlying grid, the implemented solver, and the utilized turbulence model. Most importantly, the conditions used in the computational models must replicate those of the experimental investigations as closely as possible. These include pump geometry, fluid properties such as density and viscosity, and boundary conditions like flow rate, pressure head, and rotational velocity. The magnetic levitation (MagLev) employed in the newest generation of VADs adds another layer of complexity to aligning experimental measurements and numerical models. This technology supports the rotor without mechanical fixation by axles and bearings, thereby introducing additional degrees of freedom for the rotor’s position and rotation. This constitutes a rarely addressed source of uncertainty.

Fraser et al. investigated the axial displacement of the rotor in the CentriMag at different flow rates and its impact on computational results [15]. They found that the rotor position at 3000 rpm was approximately 2.5 mm lower than at 5000 rpm, notably impacting the fluid dynamic forces computed in CFD. Movement in other directions, such as lateral shifting or tilting of the rotational axis, has not been investigated. Each rotor blade undergoes cyclic loading and unloading throughout its revolution, which could destabilize the rotational motion of the rotor. However, the general assumption for CFD is that of an ideal rotation.

Our study addresses two primary points. First, it investigates the potential of using large eddy simulations (LES) as an alternative to experimental flow field quantifications for validating RANS models of blood pumps. We conducted PIV measurements on a commercially available blood pump with magnetic levitation and modeled the same pump using LES as well as unsteady RANS with three different turbulence models. By precisely replicating the operating conditions and fluid properties of the experimental setup in the computational models, we eliminated one level of uncertainty that arises when both experiment and simulation try to replicate predefined conditions.

Second, this study examines the extent of non-rotatory impeller motion in a MagLev VAD and its impact on the flow field, addressing a potential source of discrepancy between experimental and CFD setups that has not been investigated previously. We developed a novel methodology to optically assess the three-dimensional (3D) motion of the magnetically levitated rotor within a PIV setup. By quantifying the impeller motion in 3D and modeling the main modes of the non-ideal rotation, we analyzed its impact on the overall flow field.

Our data demonstrate that while the rotor motion in the investigated commercial MagLev blood pump contains non-rotatory components, their magnitudes are small enough to be negligible for computational studies. Furthermore, we show that if all boundary conditions and fluid properties are matched, the differences between LES and PIV measurements are within the reproducibility range of PIV data and smaller than the deviation of a state-of-the-art RANS model from LES. More importantly, the areas with the largest differences between LES and PIV are more likely influenced by measurement inaccuracies than errors in the model prediction.

## Materials and Methods

All experimental and numerical investigations were conducted on the CentriMag blood pump [Thoratec Switzerland GmbH (part of Abbott), Zurich, Switzerland]. The operating condition was selected to represent left ventricular support without extracorporeal membrane oxygenation. For the experimental flow measurements, an acrylic replica of the pump housing allowing full visual access to the fluid domain was developed. The rotor was also replaced with an acrylic replica that included fluorescent markers for reconstruction of the rotor’s motion in 3D. In the computational study, two different grids were employed for the LES and unsteady RANS (uRANS) simulations, and three different uRANS closure models were implemented. In the analysis, we compared the experimental and computational phase-averaged flow fields for three different rotor blade positions, with any one of the main blades at 0°, 30°, or 60°.

### Experimental Study

#### Housing and Impeller Replica

The CentriMag is designed to allow for easy exchange of all blood-contacting components. The impeller consists of two injection-molded parts and a permanent magnet, while the housing is composed of another two injection-molded parts assembled with the impeller inside and mounted to the drive unit with a clamping mechanism. This design allowed us to replace the entire housing and impeller assembly with replicas milled out of PMMA, providing optical access to the entire domain.

The geometries of the impeller and housing were reconstructed from optical 3D scans in a previous study [16]. Based on these reconstructions, we designed an adapted housing that replicated the inner geometry of the original pump, while the outside surfaces were kept planar and normal to the laser sheet and camera axis to minimize optical refraction. The housing design also included mounting points for pressure sensors at the inlet and outlet, two reference grids for calibration of the camera view with respect to a reference frame fixed to the geometry, and grooves on the lateral faces to align the laser sheet. Each reference grid consisted of 16 drill holes with a 0.5 mm diameter, filled with two-component epoxy (resin 107106 and hardener 100149, R&G Faserverbundwerkstoffe GmbH, Waldenbuch, Germany) dyed with 0.2 weight percent (wt%) Rhodamine B (Chemie Brunschwig AG, Basel, Switzerland). These were oriented perpendicular to the laser plane to appear as points in the PIV footage. The alignment grooves were 0.5 mm high, 5 mm long notches in the opposing faces of the lower housing half that faced toward and away from the laser sheet. Four such notches at each corner were staggered at distances of 1.75, 2.25, 2.75, and 3.25 mm below the middle plane of the housing. Supplementary Fig. S1 shows the bottom half of the pump housing with the alignment grooves and calibration grids.

**Fig. 1:**
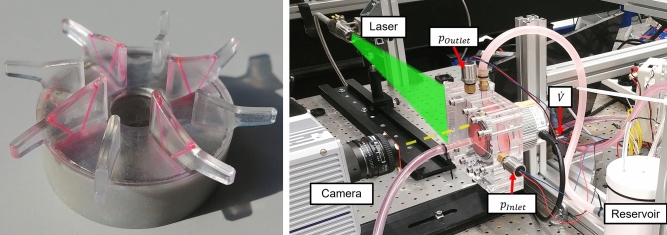
Left: Impeller replica milled out of PMMA with fluorescent channels in the main blades. Right: Experimental setup showing the camera, laser, and the main parts of the flow loop including the housing replica mounted to the original drive unit, the pressure sensors at the in- and outlet (providing *p*_inlet_ and *p*_outlet_, respectively), the flow rate sensor ($\dot{V}$), and the reservoir

The impeller replica (see Fig. 1) was designed to allow assembly with the original CentriMag magnet. The upper part, including the blades, was milled from PMMA, while the lower part holding the magnet was additively manufactured from Accura Xtreme (3D Systems GmbH, Moerfelden-Walldorf, Germany). Two counter-angled 0.5 mm holes were drilled into each of the four main blades and filled with the same fluorescently dyed epoxy used in the calibration grids. The fluorescent markings in the blades were used to determine the position of the impeller (see Section *Optical Measurement of Impeller Position*). The accuracy of the machined replicas was assessed by scanning the internal housing geometry and assembled impeller using an ATOS Compact Scan metrology system (Carl Zeiss GOM Metrology GmbH, Braunschweig) and comparing the 3D scans with the CAD model.

#### Flow Loop

For the operation of the pump during measurements, a simple flow loop was used, consisting of the pump replica connected to a fluid reservoir (see Fig. 1). The reservoir was connected to the pump inlet with PVC tubing, and the pump outlet back to the reservoir with silicone tubing. A screw compressor tubing clamp was placed on the silicone tubing to control the outflow resistance and thereby the pressure head and flow rate at a given rotational speed. The original CentriMag drive unit and console were used to operate the pump. Inlet and outlet pressures were monitored using two pressure transducers (Type 528, Huba Control, Würenlos, Switzerland) mounted on the pressure ports drilled in the housing replica. The pump flow rate was monitored using an ultrasonic flow sensor (Sonoflow CO.55, SONOTEC GmbH, Halle, Germany) clamped on the PVC tubing. The flow rate sensor had been previously calibrated with the employed tubing and testing liquid.

As the testing liquid, a mixture of 28.1 wt% water, 24.4 wt% glycerol, and 47.5 wt% ammonium thiocyanate was prepared to achieve a viscosity in the physiological range of blood and match the refractive index of PMMA. The refractive index of the final solution was fine-tuned to 1.493. The density of the final testing liquid was 1164 kg/m^3^. The viscosity was measured using a double-gap system on a rheometer (MCR302, Anton Paar, Buchs, Switzerland) over temperatures ranging from 24 to 31 °C, and a linear relationship was derived:

1 $$\mu = \mu_{0} + C_{T} \cdot T$$

where $\mu$ is the dynamic viscosity in Pa·s, $T$ is the temperature in °C, and $\mu_{0} = 5.975 \cdot 10^{ - 3}$ Pa·s and $C_{T} = - 8.55 \cdot 10^{ - 5}$ Pa·s/°C are model parameters adjusted to fit the experimental data. The average temperature of the testing liquid during the measurements was 24.3 °C, yielding a testing liquid viscosity of 0.0039 Pa⋅s.

#### PIV Setup

The PIV setup consisted of a diode-pumped Nd-YLF laser (Quantronix, Darwin Duo 527 nm, USA) to illuminate the flow field and a high-speed camera (Photron SA5, Japan) equipped with AF Micro Nikkor 28 mm and 60 mm f2.8D lenses (Nikon, Japan) to capture the images. Fluorescent polyethylene microspheres (Cospheric, Santa Barbara, USA), with a diameter range of 63–75 µm and a density of 1.00 g/ml, were used as tracer particles. Their excitation wavelength was at 575 nm and their emission wavelength at 607 nm. An orange band-pass filter with a wavelength of 514 nm was used to isolate the emitted light. The laser sheet was aligned with the grooves in the housing 2.25 mm below the middle plane.

The recordings were performed with 10,000 frames per second with a resolution of 896x848 pixels. A laser sheet with a thickness of 0.5 mm was created using optical lenses to illuminate the region of interest. Two different views were recorded: To capture sufficient blade markers for the impeller motion reconstruction, the entire impeller domain was recorded using the 28 mm lens, while a close-up of the outlet quadrant with the 60 mm lens was used for the PIV measurements. Raw footage of both recordings is available on Zenodo [17]. Given the camera resolution and observed domain, the spatial resolution for the PIV measurements was approximately 60 µm/px. 

#### PIV Analysis and Postprocessing

Computation of the PIV velocity fields was performed using the PIVlab toolbox [18, 19] in MATLAB (Mathworks Inc., Natick, Massachusetts, USA). The settings used are listed in the supplementary material. Since PIVlab only allows for uniform spatial calibration, velocities were initially calculated without calibration in their raw pixels-per-frame units within PIVlab and then exported for further processing in Matlab. Spatial calibration of the raw PIV velocity field was based on the calibration grid implemented in the acrylic housing and fine-tuned by matching the boundaries of the fluid domain in the PIV section to the pump internal boundaries in the CAD geometry. After calibration, the PIV data were interpolated on a uniform Eulerian grid with a resolution of 0.1 mm in $\mathop{X}\limits^{\rightharpoonup}$ and $\mathop{Y}\limits^{\rightharpoonup}$ .Temporal calibration was based on the known imaging frequency of 10,000 frames per second (fps).

Phases for phase-averaging the PIV data were classified algorithmically based on the angular position of the impeller, which was determined for each PIV image pair using the fluorescent blade markings as described in Section *Optical Measurement of Impeller Position*. Blade positions were then sorted into 1-degree bins from 1° to 90°. This sorting was done modulo 90°, accounting for the impeller’s fourfold rotational symmetry. For each angular position, the averaged in-plane velocity components were computed over all available instantaneous velocity fields for that angular position. For the blade positions evaluated in this manuscript (0°, 30°, and 60°), sets of 228, 284, and 251 velocity fields were available, respectively. The phase-averaged in-plane velocity magnitude $\overline{U}_{i}^{\alpha }$ was then calculated from these phase-averaged velocity components as

2 $$\overline{U}_{i}^{\alpha } = \sqrt { \left( {\overline{{u_{X} }}_{ i}^{ \alpha } } \right)^{2} + \left( {\overline{{u_{Y} }}_{ i}^{ \alpha } } \right)^{2} }$$

where α is the angular position of the rotor, $i \in \left[ {1, \ldots ,N} \right]$ is the grid node index with $N$ being the number of nodes in the observed domain, and $\overline{{u_{X} }}_{ i}^{ \alpha }$ and $\overline{{u_{Y} }}_{ i}^{ \alpha }$ are the phase-averaged velocity components in $\mathop{X}\limits^{\rightharpoonup}$ and $\mathop{Y}\limits^{\rightharpoonup}$ .

For comparison between two different phase-averaged velocity fields (e.g., PIV and CFD), the local relative error was computed in line with the definition in the FDA benchmark study [9]:

3 $${\rm E}_{rel,i} \left( {\overline{U}^{\alpha } } \right) = \frac{{\overline{U}_{i}^{\alpha } - \overline{U}_{ref,i}^{\alpha } }}{{\max \left( {\overline{U}_{ref,i}^{\alpha } } \right)}} ,$$

where $\overline{U}_{ref,i}^{\alpha }$ is the reference velocity field, and $\overline{U}_{i}^{\alpha }$ the field for which the deviation to the reference is to be computed. To quantify the overall error within the observed field, the root-mean-square error (RMS) was also computed in line with the FDA benchmark study:

4 $$RMS = 100\sqrt {\frac{1}{N}\mathop \sum \limits_{i = 1}^{N} \left( {{\rm E}_{rel, i} } \right)^{2} }$$

For comparison of pressure fields, pressure was evaluated as relative pressure to the inlet and the local absolute error was used instead of the relative one, defined as:

5 $${\rm E}_{abs,i} \left( {\overline{p}^{\alpha } } \right) = \overline{p}_{i}^{\alpha } - \overline{p}_{ref,i}^{\alpha }$$

where $\overline{p}$ is the phase-averaged pressure, relative to the pressure at the inlet boundary.

#### Optical Measurement of Impeller Position

Optical three-dimensional position or motion measurements are commonly carried out using stereoscopic imaging, which requires at least two cameras to reconstruct depth information through triangulation. In this study, we implemented an alternative approach that enables full 3D reconstruction of the impeller position and orientation using only a single-camera view. Depth information was recovered from strategically placed fluorescent marker channels in the main blades of the impeller replica. These markers allowed us to infer the impeller’s motion using the same setup as for PIV, with only two modifications: the camera lens was replaced with one of 28 mm focal length to expand the field of view, and the fluid was left unseeded to improve marker visibility. The following paragraphs describe how impeller position and orientation were reconstructed from the single-camera view, based on the spatial configuration of the fluorescent blade markers.

**Fig. 2:**
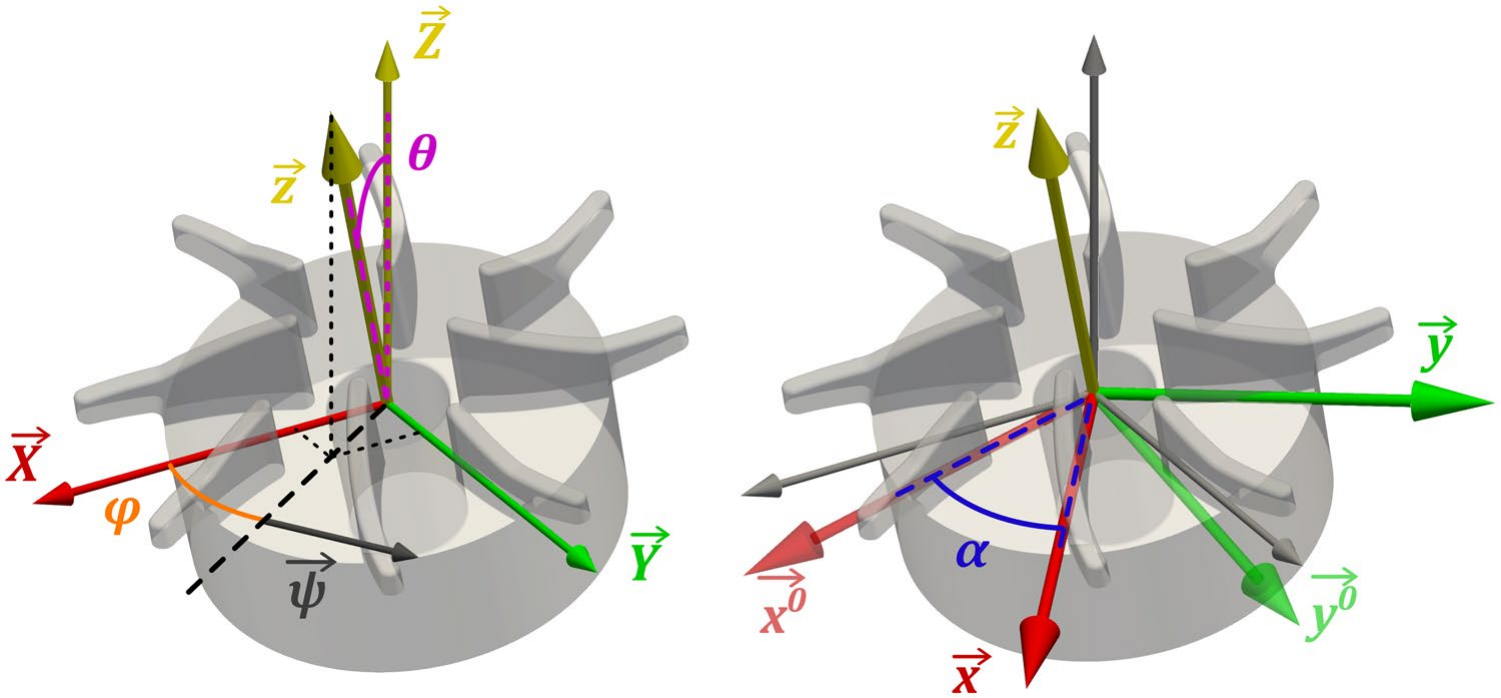
Definition of the rotor position and alignment in 3D. $$\mathop{X}\limits^{\rightharpoonup}$$, $$\mathop{Y}\limits^{\rightharpoonup}$$, and $$\mathop{Z}\limits^{\rightharpoonup}$$ are the axes of the fixed global coordinate system. $$\mathop{x}\limits^{\rightharpoonup}$$ , $$\mathop{y}\limits^{\rightharpoonup}$$, and$$\mathop{z}\limits^{\rightharpoonup}$$ are the axes of the coordinate system fixed to the rotor center, where $$\mathop{z}\limits^{\rightharpoonup}$$ is aligned with the rotor axis and $$\mathop{x}\limits^{\rightharpoonup}$$ is aligned with the leading edge of the first main blade. $$\varphi$$ (azimuth) and $$\theta$$ (polar angle) indicate the position of the rotor axis in spherical coordinates. Finally, $${\upalpha }$$ indicates the angular position around $$\mathop{z}\limits^{\rightharpoonup}$$ relative to $$\overset{\lower0.5em\hbox{$\smash{\scriptscriptstyle\rightharpoonup}$}}{{{\mathrm{x}}^{0} }}$$ and $$\overset{\lower0.5em\hbox{$\smash{\scriptscriptstyle\rightharpoonup}$}}{{{\mathrm{y}}^{0} }}$$ , which represents the pure tilt of $$\mathop{X}\limits^{\rightharpoonup}$$ and $$\mathop{Y}\limits^{\rightharpoonup}$$ without further rotation. Not represented here is the possibility of a translation between the global and local coordinate systems where the origin of $$\left( {\mathop{x}\limits^{\rightharpoonup} ,\mathop{y}\limits^{\rightharpoonup} ,\mathop{z}\limits^{\rightharpoonup} } \right)$$ does not fall on the origin of $$\left( {\mathop{X}\limits^{\rightharpoonup} ,\mathop{Y}\limits^{\rightharpoonup} ,\mathop{Z}\limits^{\rightharpoonup} } \right)$$

At any given moment, the position of the impeller is uniquely defined by the orientation of its central axis, the angular position of the first main blade around this axis and the position of its center, $OR$, defined as the intersection between the impeller’s central axis and the reference plane, i.e., the plane aligned with the bottom of the blades (Fig. 2). All these entities were derived from the blade channel markings as summarized below and detailed in the supplement.

For the subsequent derivations, we define two coordinate systems as depicted in Fig. 2: a fixed global coordinate system $\left( {\mathop{X}\limits^{\rightharpoonup} ,\mathop{Y}\limits^{\rightharpoonup} ,\mathop{Z}\limits^{\rightharpoonup} } \right)$ referenced to the mid-plane of the two housing halves and a local coordinate system $\left( {\mathop{x}\limits^{\rightharpoonup} ,\mathop{y}\limits^{\rightharpoonup} ,\mathop{z}\limits^{\rightharpoonup} } \right)$ anchored to the impeller. The origin of the global coordinate system, $OG$, is at the intersection between the central axis of the inlet cannula and the symmetry plane of the volute region of the housing. The $\mathop{Z}\limits^{\rightharpoonup}$ axis is congruent to the central axis of the inlet cannula, while the $\mathop{X}\limits^{\rightharpoonup}$ axis is parallel to the central axis of the outlet cannula. The origin of the local coordinate system is at the center of the rotor, $OR$. The $\mathop{z}\limits^{\rightharpoonup}$ axis is aligned with the impeller’s central axis, while the $\mathop{x}\limits^{\rightharpoonup}$ axis is aligned with the leading edge of the first main blade and rotates with it.

Let $R_{{\mathop{n}\limits^{\rightharpoonup} }} \left( {\Omega } \right)$ be the transformation matrix for a rotation of angle ${\Omega }$ around the axis $\mathop{n}\limits^{\rightharpoonup}$. The correspondence between local and global coordinates of a point $P$ can then be formalized as

6 $$\begin{gathered} \left( {\begin{array}{*{20}c} {X_{P} } \\ {Y_{P} } \\ {Z_{P} } \\ \end{array} } \right) = \left( {\begin{array}{*{20}c} {X_{OR} } \\ {Y_{OR} } \\ {Z_{OR} } \\ \end{array} } \right) + R_{{\mathop{z}\limits^{\rightharpoonup} }} \left( \alpha \right) \cdot R_{{\mathop{\psi }\limits^{\rightharpoonup} }} \left( \theta \right) \cdot \left( {\begin{array}{*{20}c} {x_{P} } \\ {y_{P} } \\ {z_{P} } \\ \end{array} } \right), \\ {\mathrm{with}} \mathop{\psi }\limits^{\rightharpoonup} = R_{{\mathop{Z}\limits^{\rightharpoonup} }} \left( \varphi \right) \cdot \mathop{Y}\limits^{\rightharpoonup} \hfill {\text{and }} \mathop{z}\limits^{\rightharpoonup} = R_{{\mathop{\psi }\limits^{\rightharpoonup} }} \left( \theta \right) \cdot \mathop{Z}\limits^{\rightharpoonup}, \hfill \\ \end{gathered}$$

where the direction of the rotor axis, $$\mathop{z}\limits^{\rightharpoonup}$$, in the global coordinate system is given by $$\varphi$$ (azimuth) and $$\theta$$ (polar angle) (Fig. 2) and the impeller center by $$\left( {X_{OR} ,Y_{OR} ,Z_{OR} } \right)$$. The vector $$\mathop{\psi }\limits^{\rightharpoonup}$$ is normal to the projection of the impeller’s central axis $$\mathop{z}\limits^{\rightharpoonup}$$ onto the global ($$\mathop{X}\limits^{\rightharpoonup} , \mathop{Y}\limits^{\rightharpoonup} )$$ plane. $$R_{{\mathop{z}\limits^{\rightharpoonup} }} \left( {\upalpha } \right)$$ describes the rotation of the impeller around its axis by $$\alpha$$ and $$R_{{\mathop{\psi }\limits^{\rightharpoonup} }} \left( \theta \right)$$ the rotation around $$\mathop{\psi }\limits^{\rightharpoonup}$$ by the magnitude of $$\theta$$. If applied to the rotor in an arbitrary position $$R_{{\mathop{z}\limits^{\rightharpoonup} }} \left( {\upalpha } \right)$$ would re-align $$\mathop{x}\limits^{\rightharpoonup}$$ with $$\overset{\lower0.5em\hbox{$\smash{\scriptscriptstyle\rightharpoonup}$}}{{x_{0} }}$$ and $$R_{{\mathop{\psi }\limits^{\rightharpoonup} }} \left( \theta \right)$$ would set it upright, aligning $$\mathop{z}\limits^{\rightharpoonup}$$ with $$\mathop{Z}\limits^{\rightharpoonup}$$.

**Fig. 3:**
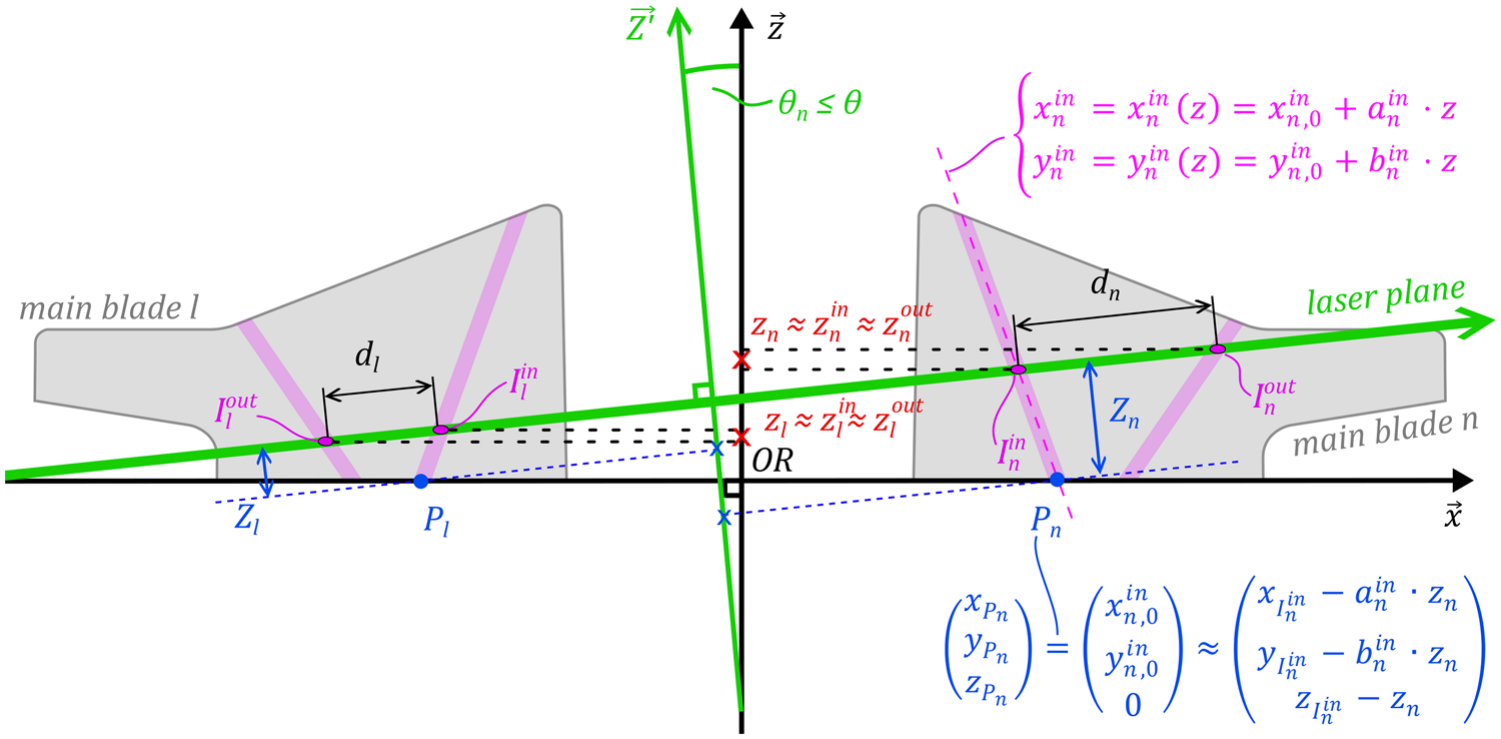
Illustration of the derivation of the global coordinates of the reference point $$P_{{\mathrm{n}}}$$, defined as the intercept of the inner marker channel and the rotor reference plane. The procedure is illustrated here for the first and third main blade, aligned with the local $$\mathop{x}\limits^{\rightharpoonup}$$ axis. As the marker channels of opposing blades do not lie on a common plane due to the curvature of the blades, this depiction is simplified as it only shows the projected view on the ($$\mathop{x}\limits^{\rightharpoonup} ,\mathop{z}\limits^{\rightharpoonup}$$) plane. $$I_{n}^{in}$$ and $$I_{n}^{out}$$ lie at the intersection between the inner and outer marker channels and the laser plane and appear as two illuminated dots in the camera images. $$\overset{\lower0.5em\hbox{$\smash{\scriptscriptstyle\rightharpoonup}$}}{Z^{\prime}}$$ is the projection of the global axis $$\mathop{Z}\limits^{\rightharpoonup}$$ in the plane of the blades under consideration (here the ($$\mathop{x}\limits^{\rightharpoonup} ,\mathop{z}\limits^{\rightharpoonup}$$) plane). $${\theta }_{{\mathrm{n}}}$$ is the angle between the laser sheet and impeller reference plane as seen from that cut plane and verifies $$\theta_{n} \le \theta$$. In the current example with an ($$\mathop{x}\limits^{\rightharpoonup} ,\mathop{z}\limits^{\rightharpoonup}$$) cut-plane, $$\theta_{n}$$ is such that $$\left| {\tan \left( {\theta_{n} } \right)} \right| = \left| {\cos \left( \varphi \right)\tan \left( \theta \right)} \right| \le \left| {\tan \left( \theta \right)} \right|$$. The global coordinates $$\left( {{\mathrm{X}}_{{{\mathrm{P}}_{{\mathrm{n}}} }} ,{\mathrm{Y}}_{{{\mathrm{P}}_{{\mathrm{n}}} }} ,{\mathrm{Z}}_{{{\mathrm{P}}_{{\mathrm{n}}} }} } \right)$$ can be derived from $$\left( {x_{{P_{n} }} ,y_{{P_{n} }} ,z_{{P_{n} }} } \right)$$ using (12). The supplement shows an additional view along $$\mathop{z}\limits^{\rightharpoonup}$$ next to a recorded frame, showing the illuminated markings

Each of the four main blades of the rotor replica features two non-parallel embedded fluorescent channels drilled straight through the blade, an inner (in) and an outer (out) one. The base-positions and orientations of the channels are described by the parameters $x_{n,0}$ ,$y_{n,0}$ and $a_{n}$ and $b_{n}$ , respectively (see Fig. 3), where $n \in \left\{ {1, 2, 3, 4} \right\}$ is the number of the respective blade. Hence, any position $\left( {x, y} \right)$ on a channel centerline is uniquely defined by the height $z$ from the rotor plane and the main blade n it belongs to:

7 $$\left\{ {\begin{array}{*{20}c} {x_{n}^{out} = x_{n}^{out} \left( z \right) = x_{n,0}^{out} + a_{n}^{out} \cdot z} \\ {y_{n}^{out} = y_{n}^{out} \left( z \right) = y_{n, 0}^{out} + b_{n}^{out} \cdot z} \\ \end{array} } \right.$$

8 $$\left\{ {\begin{array}{*{20}c} {x_{n}^{in} = x_{n}^{in} \left( z \right) = x_{n,0}^{in} + a_{n}^{in} \cdot z} \\ {y_{n}^{in} = y_{n}^{in} \left( z \right) = y_{n, 0}^{in} + b_{n}^{in} \cdot z} \\ \end{array} } \right.$$

In the camera view, the intersection between a channel and the laser plane appears as an ellipse (see camera view in supplementary Fig. S2b). The center points of the inner and outer channel ellipses of main blade $n$, $I_{n}^{in}$, and $I_{n}^{out}$, are separated by the distance $d_{n}$. It can be expressed as a function of the heights $z_{n}^{in}$ and $z_{n}^{out}$ at which the laser plane intersects the inner and outer channels, respectively:

9 $$d_{n} \left( {z_{n}^{in} , z_{n}^{out} } \right) = \sqrt {\left( {x_{n}^{out} \left( {z_{n}^{out} } \right) - x_{n}^{in} \left( {z_{n}^{in} } \right)} \right)^{2} + \left( {y_{n}^{out} \left( {z_{n}^{out} } \right) - y_{n}^{in} \left( {z_{n}^{in} } \right)} \right)^{2} + \left( {z_{n}^{out} - z_{n}^{in} } \right)^{2} }$$

Combining equations (7)-(9) and neglecting the height difference between $z_{n}^{in} \approx z_{n}^{out} = z_{n}$ (see supplement), we obtain

10 $$d_{n}^{2} = \left( {\Delta a_{n} \cdot z_{n} + \Delta x_{n,0} } \right)^{2} + \left( {\Delta b_{n} \cdot z_{n} + \Delta y_{n,0} } \right)^{2}$$

with ${\Delta }x_{n,0} = x_{n,0,out} - x_{n,0,in}$ , ${\Delta }y_{n,0} = y_{n,0,out} - y_{n,0,in}$ , ${\Delta }a_{n} = a_{n,out} - a_{n,in}$ and ${\Delta }b_{n} = b_{n,out} - b_{n,in}$. Of the two roots of this quadratic equation, only the one yielding positive height values for the channel centerlines is of relevance:

11 $$\begin{aligned} & z_{n} = \frac{{ - \left( {\Delta x_{n,0} \cdot \Delta a_{n} + \Delta y_{n,0} \cdot \Delta b_{n} } \right) + \sqrt \delta }}{{\left( {\Delta a_{n} } \right)^{2} + \left( {\Delta b_{n} } \right)^{2} }}, \\& \text{ with } \delta = (\Delta x_{n,0} \cdot \Delta a_n + \Delta y_{n,0} \cdot \Delta b_n )^2+(\Delta a_n^2 + \Delta b_n^2 ) \cdot (d_n^2-d_{n,0}^2 ) \end{aligned}$$

where $d_{n,0} = \sqrt {{\Delta }x_{n,0}^{2} + {\Delta }y_{n,0}^{2} }$. Plugging (11) into (8), and inserting the global coordinates of $I_{n}^{in}$ gained by optical measurement, we obtain the global coordinates of the intercept between the inner marker channel centerline and the impeller reference plane:

12 $$\begin{gathered} P_{n} = \left( {\begin{array}{*{20}c} {X_{{I_{n}^{in} }} } \\ {Y_{{I_{n}^{in} }} } \\ {Z_{{I_{n}^{in} }} } \\ \end{array} } \right) - R_{{\mathop{Z}\limits^{\rightharpoonup} }} \left( \alpha \right) \cdot R_{{\mathop{\psi }\limits^{\rightharpoonup} }} \left( \theta \right) \cdot \left( {\begin{array}{*{20}c} {a_{n}^{in} \cdot z_{n} } \\ {b_{n}^{in} \cdot z_{n} } \\ {z_{n} } \\ \end{array} } \right) \\ \approx \left( {\begin{array}{*{20}c} {X_{{I_{n}^{in} }} } \\ {Y_{{I_{n}^{in} }} } \\ {Z_{{I_{n}^{in} }} } \\ \end{array} } \right) - R_{{\mathop{Z}\limits^{\rightharpoonup} }} \left( \alpha \right) \cdot \left( {\begin{array}{*{20}c} {a_{n}^{in} \cdot z_{n} } \\ {b_{n}^{in} \cdot z_{n} } \\ {z_{n} } \\ \end{array} } \right) \end{gathered}$$

The simplification at the end of (12) is based on the same argumentation used to simplify $z_{n}^{in} \approx z_{n}^{out} = z_{n}$ (see supplement).

Using images in which the markers of at least three blades $n,m$ and $l \in \left\{ {1,2,3,4} \right\}$ (with $n \ne m \ne l$) are visible and therefore the position of three points $P_{n}$, $P_{m}$ and $P_{l}$ on the impeller reference plane can be computed, we can derive the vector of the rotor axis in global coordinates $\overrightarrow {{z^{G} }}$ through the orthonormal basis of the vectors spanned between the three points:

13 $$\overrightarrow {{z^{G} }} = \overrightarrow {{P_{n} P_{m} }} \times \overrightarrow {{P_{n} P_{l} }}$$

Additionally, the center of the impeller can be computed by taking the mid-point between the two opposing blade’s reference points, namely $P_{{{\mathrm{center}}}} = \frac{1}{2}\left( {P_{n} + P_{l} } \right)$ .

### Numerical Study

#### Geometry and Mesh

**Fig. 4:**
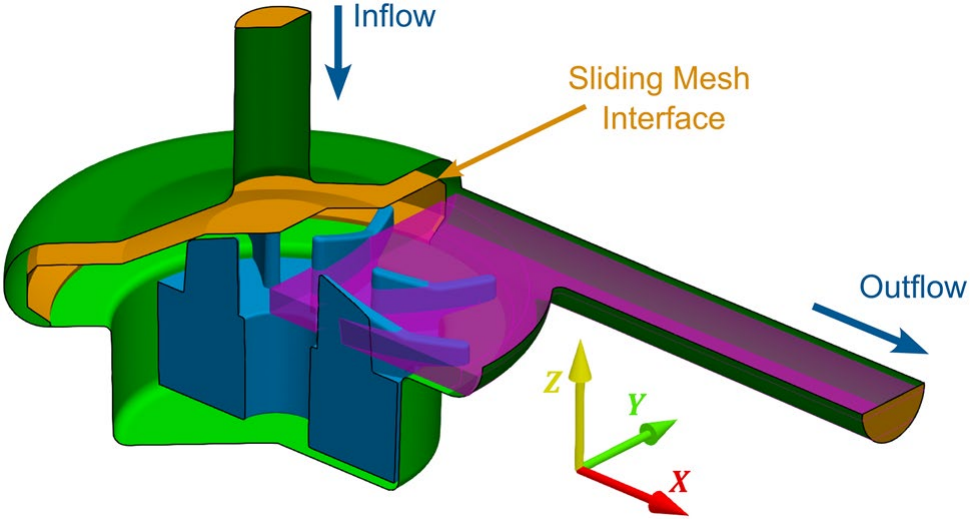
Geometry of the CentriMag pump, including computational interfaces and the volume slice that was probed for postprocessing. Inlet and outlet extensions are not depicted. Dark and light green: surface of the pump housing in the volute and impeller region, respectively. Blue: Impeller surface. Orange: Numerical interfaces. A sliding mesh interface separates the fixed volute and rotating impeller domains. The two other orange surfaces interface the inlet and outlet cannulas with their extensions. Magenta: Volume slice that encompasses all cells falling into the observational region of the PIV measurements. This volume slice was probed at every time-step to collect time-resolved data for postprocessing

The fluid domain, defined by the boundaries of the housing and rotor walls, was divided into a volute region and a rotor region, separated by a manually constructed interface (orange surface in Fig. 4). To avoid regional separation within the small radial gap between rotor and housing, the interface was terminated perpendicular to the housing wall, before entering the gap. To reduce boundary effects, the inlet cannula was extended by 50 mm (diameter: 9.2 mm) and the outlet by 250 mm (diameter: 9.5 mm). The position of the rotor was set with the rotor axis coincident with the global $\mathop{Z}\limits^{\rightharpoonup}$ axis and with the rotor reference plane at Z = − 5.15 mm to replicate the average of the optically measured Z position. For simulations of non-ideal rotation, the impeller was made to precess around the globa﻿l $\mathop{Z}\limits^{\rightharpoonup}$ axis with a 1° tilt angle.

Two different grids comprising around 13.5 million and 150 million cells were used for the uRANS and LES models, respectively. Mesh parameters, grid-refinement studies for the uRANS setup, and validation of the LES based on power spectra and a global energy balance are described in [20]. For both models, the volute and impeller regions were discretized with a polyhedral grid in the fluid core and boundary layer-resolving prism layers along all walls. Prism layer parameters were set to ensure that the non-dimensional normal wall-distance $y^{ + }$ was < 1. For the LES grid, the surface resolution was set to twice the height of the near-wall prism layer to minimize the aspect ratio of all prism layers. The resulting non-dimensional tangential wall-distances in the LES grid were well below the recommended limit of 15 [21].

#### Flow Models

All simulations were performed using the finite volume CFD software Siemens Star-CCM+ (Siemens, Munich, Germany). Fluid properties were matched to those of the experimental blood-analog with a dynamic viscosity of 0.0039 Pa·s and a density of 1164 kg/m^3^. In the LES, turbulence was modeled using the wall-adapting local-eddy viscosity (WALE) sub-grid-scale model, while in the uRANS setups, k-ε EB, k-ω SST, and RST turbulence models were used as implemented in Star-CCM + .

Spatial discretization was implemented using a second-order bounded central scheme with an upwind blending factor of 0.15 for the LES and a second-order upwind scheme for uRANS. Time derivatives were discretized using an implicit second-order scheme in all cases. The LES required a time-step size equivalent to 0.125° to ensure a CFL number < 1 throughout the domain and to limit the motion of the sliding volute-impeller interface to less than one cell size per time-step. For uRANS, a time-step size equivalent to 1° of rotation was sufficient to achieve these requirements. Inner iterations were stopped when the mass flow across the volute-impeller interface fell below $10^{ - 8}$ kg/s and the relative change in inlet pressure stayed below $10^{ - 6}$ over the previous 5 inner iterations. These entities were chosen to define stopping criteria because they were generally the last to converge.

#### Boundary Conditions and Flow Regime

Boundary conditions were chosen to represent the experimental settings of 4.4 L/min flow rate at 2,350 rpm. To obtain velocity profiles and turbulence metrics representative of a fully developed flow, a separate simulation setup was used. By considering a short, straight section of the inlet tubing with periodic boundary conditions at in- and outlet and a target volume flow rate of 4.4 L/min, the flow in an infinitely long tube was modeled and the resulting velocity and turbulence metrics profile across the tube were used as boundary condition for the inlet. Details of this process are described in [20]. The outlet boundary was set to an average pressure of 0 Pa, while turbulence intensity $I_{turb}$ and turbulent length scale $l_{turb}$ were estimated according to (14) and (15) for fully developed pipe flow:

14 $$I_{turb} = 0.16 \cdot {Re}^{{ - \frac{1}{8}}}$$

15 $$l_{turb} = 0.07 \cdot D_{h} ,$$

where $Re$ is the Reynolds number and $D_{h}$ is the hydraulic diameter.

The impeller rotation was modeled as a rigid body motion of the entire impeller region, utilizing a sliding mesh interface between the impeller and the volute domain. Given that the impeller region encompasses the walls of the static housing (depicted as the light green surface in Fig. 4), a counter-rotating velocity distribution was applied to them, thereby achieving zero net absolute velocity along the walls.

#### Initialization and Data Collection

The LES case was initialized with the converged fields of the LES simulation from a previous study [20], which only differed with a slightly lower impeller position and slightly different fluid density. It was run for three rotations to allow for adjustment of the flow field to the new conditions. Afterward, data were collected for twelve rotations at which point the mean and variance of the recorded metrics had reached statistical convergence.

All uRANS cases were initialized with the converged fields of a precursor simulation using the k-ε EB setup and had been run for ten rotations to converge to a statistical steady state. They were then run for ten rotations to stabilize before data tracking was started. Data were then collected for ten rotations.

During data collection, the velocity components and pressure in all cells within the Z-slice of the outlet quadrant (as depicted in Fig. 4) were extracted for every 30° of rotation. The resulting raw volumetric data is available on Zenodo for the LES and all uRANS setups [22, 23].

#### Postprocessing

The data extracted from the LES and uRANS cases was interpolated onto the Eulerian PIV grid. This grid had uniform in-plane resolution of 0.1 mm ($\mathop{X}\limits^{\rightharpoonup}$ and $\mathop{Y}\limits^{\rightharpoonup}$ directions), and through-plane resolution of 0.125 mm ($\mathop{Z}\limits^{\rightharpoonup}$-direction). The interpolated data were then phase-averaged for impeller positions 0°, 30° and 60° with each recorded rotation providing 4 instances for each impeller position (due to the fourfold rotational symmetry of the impeller). 0° was defined as the leading edges of the four blades lying on the $\mathop{X}\limits^{\rightharpoonup}$ and $\mathop{Y}\limits^{\rightharpoonup}$ axes of the global coordinate system.

The phase-averaged planar velocity magnitude $\overline{U}_{i}^{\alpha }$ was computed according to (2). For comparison with PIV data, $\overline{U}_{i}^{\alpha }$ was then averaged over Z in the range covered by the laser sheet (−2.5 to −2.0 mm).

## Results

### Geometrical Accuracy of CentriMag Replica

The comparison of the scanned surfaces of the milled PMMA replicas from the CAD models of housing halves and the rotor showed all parts to be highly accurate with over 99.5% of all fluid-contacting surfaces falling within ± 0.1 mm of the CAD reference. A depiction of the normal deviation between scanned surface and CAD reference is available in the supplementary material (Fig. S3). While also staying within ± 0.1 mm of the CAD reference, the rotor shows larger overall deviations than the housing. This can be explained by it being an assembled part, adding assembly tolerances to the manufacturing tolerances, and by the small thickness of the blades, which leads to deformation during the milling process and, therefore, to inaccuracies. Overall, we judged the manufacturing accuracy to be sufficiently high for the purposes of this study.

### Impeller Motion

**Fig. 5:**
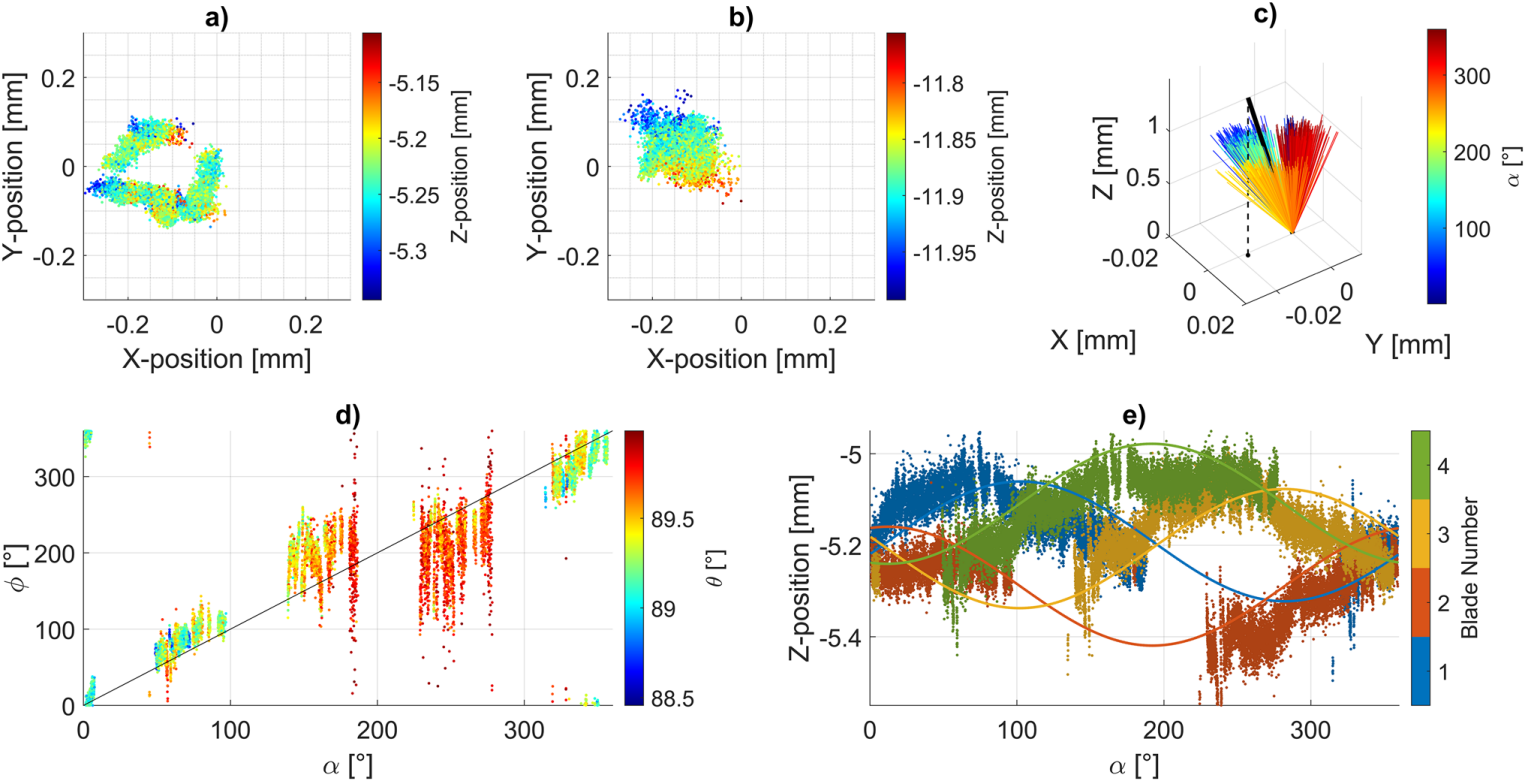
Measured impeller position and motion expressed in the global coordinate system $\left( {\mathop{X}\limits^{\rightharpoonup} ,\mathop{Y}\limits^{\rightharpoonup} ,\mathop{Z}\limits^{\rightharpoonup} } \right)$ with origin in ${\mathrm{OG}}$, at the intersection between the central axis of the inlet cannula and the symmetry plane of the volute. **a** Position of the impeller center OR. **b** Position of the derived motion center. **c** All measured impeller axis-orientations in the global coordinate system, depicted as lines of unit length, starting at the origin. The black thick line represents the overall mean (scaled by 1.5 for visibility) and its plumbline as dashed line. **d** Deviating Azimuth $\phi$ of the impeller (as defined in (16)), plotted over the azimuth of the impeller ${\upalpha }$. Hence, $\phi$ describes the azimuth of the impeller axis relative to the mean rotational axis. **e** Z-positions of reference points $P_{n}$ on the individual blades over the azimuth of the impeller as derived from the blade marker positions (dots) and as predicted based on the modeled impeller motion (line). A detailed description of the global and local coordinate systems and reference points is provided in Section *Optical Measurement of Impeller Position*

Figure 5 illustrates the reconstructed position and motion of the impeller. The impeller’s center at the height of the laser plane is clearly affected by overall impeller movement. This can be discerned in Fig. 5a, which shows the position of point OR (the center of the impeller on the impeller reference plane) in the global reference system. We determined the impeller’s center of motion by projecting the impeller center along the computed impeller axis and selecting the projection distance with the minimal standard deviation of the center in $\mathop{X}\limits^{\rightharpoonup}$ and $\mathop{Y}\limits^{\rightharpoonup}$ (Fig. 5b). While the position of OR is distributed in a toroidal pattern, the motion center’s position is constrained to a single cluster, which is shifted relative to the global origin OG. Figure 5c depicts the orientation of the impeller’s axis across the entire dataset, along with the overall mean axis. The impeller exhibits a slight but noticeable average inclination of its axis relative to the global $\mathop{Z}\limits^{\rightharpoonup}$ axis. Over time, the orientation of the impeller axis forms a conical shape around the mean axis, indicating a precessing motion. Figure 5d visualized the deviating azimuth $\phi$, which is the azimuth of the impeller axis relative to the mean axis and can be derived as the azimuth of the impeller axis after correcting for the mean tilt:

16 $$\phi \left( {\mathop{z}\limits^{\rightharpoonup} } \right) = \varphi \left( {R_{\overrightarrow{\psi}_{mean}} \left( {\theta_{mean} } \right) \cdot \mathop{z}\limits^{\rightharpoonup} } \right)$$

where the subscript $mean$ denotes the time-average across all data-points and $R_{\overrightarrow{\psi}_{mean}} \left( {\theta_{mean} } \right)$ is the rotation matrix around the mean of $\mathop{\psi }\limits^{\rightharpoonup}$ by the mean elevation $\theta_{mean}$. Figure 5d shows that $\phi$ is scattered around the identity line to the impeller phase angle, suggesting that the axis precesses in sync with the impeller rotation. The increased spread of the data between 170 and 280° of the impeller’s azimuth is attributed to the axis being nearest to the global $\mathop{Z}\limits^{\rightharpoonup}$-axis at these angles, which magnifies the impact of measurement inaccuracies in $\mathop{X}\limits^{\rightharpoonup}$ and $\mathop{Y}\limits^{\rightharpoonup}$ on the derived azimuth.

Summarizing the measurements, the impeller motion was found to deviate from an ideal rotation in a complex pattern. The main motion as recorded can be approximated by transforming an ideal rotation in three steps:

1) Shift of the impeller center (Fig. 5b): The motion center of the impeller is shifted with respect to its expected location by about −0.13 mm in $\mathop{X}\limits^{\rightharpoonup}$ and 0.04 mm in $\mathop{Y}\limits^{\rightharpoonup}$, with directions defined in Fig. 4. The standard deviations of the measured impeller center are 0.03-0.04 mm in both $\mathop{X}\limits^{\rightharpoonup}$ and $\mathop{Y}\limits^{\rightharpoonup}$, which fall below the pixel accuracy of the images.

2) Tilt of mean rotation axis (Fig. 5c): The mean rotational axis is tilted around the motion center with a polar angle of approximately 0.65° and an azimuthal angle of approximately 273°. As each blade travels along a circular path with this tilt, it sinks and rises in $\mathop{Z}\limits^{\rightharpoonup}$ over one rotation, following a sinusoidal curve with a magnitude of approximately ± 130 µm in $\mathop{Z}\limits^{\rightharpoonup}$.

3) Precession of the impeller axis around its mean (Fig. 5d): The impeller axis shows an inclination relative to the mean rotational axis of approximately 0.45°, with an azimuthal angle of approximately 275° relative to the first main blade. The inclination axis rotates around the mean rotational axis with the same frequency as the impeller, causing the same side of the impeller to always be elevated while the opposite side is lowered. 

To evaluate the accuracy of this decomposition of the impeller’s motion, we modeled the motion of the impeller’s blades using the described transformation steps. Figure 5e shows these reconstructed blade motions superimposed on the measured blade positions. While the reconstruction does not perfectly match the measured data, it captures the main modes of Z travel. Additional deviations could be caused by inaccuracies in the manufacturing of the fluorescent channels of the impeller, as well as the impact of the simplifying assumptions made in the reconstruction in II.A.5. Nevertheless, we judged our reconstruction to satisfactorily represent the extent of the impeller’s deviation from an ideal rotation, allowing us to proceed with the analysis of its impact.

To investigate the impact of the non-ideal rotation, we replicated an exaggeration of the precession motion mode described under point 3 above, as we judged this to be the most intrusive. We implemented the precession motion by tilting the impeller within the rotating region of the CFD model but keeping the rotation axis of the rotating region vertical. We selected a tilt magnitude of 1°, which represents the upper bound of the measured inclination of the impeller plane at any point in time. Supplementary Fig. S4 illustrates the impact of this 1° tilt on the impeller position. Supplementary Fig. S5 provides close-up views of the mesh in the tilted configuration, highlighting the relative scale of the positional deviation compared to the local mesh size.

**Fig. 6:**
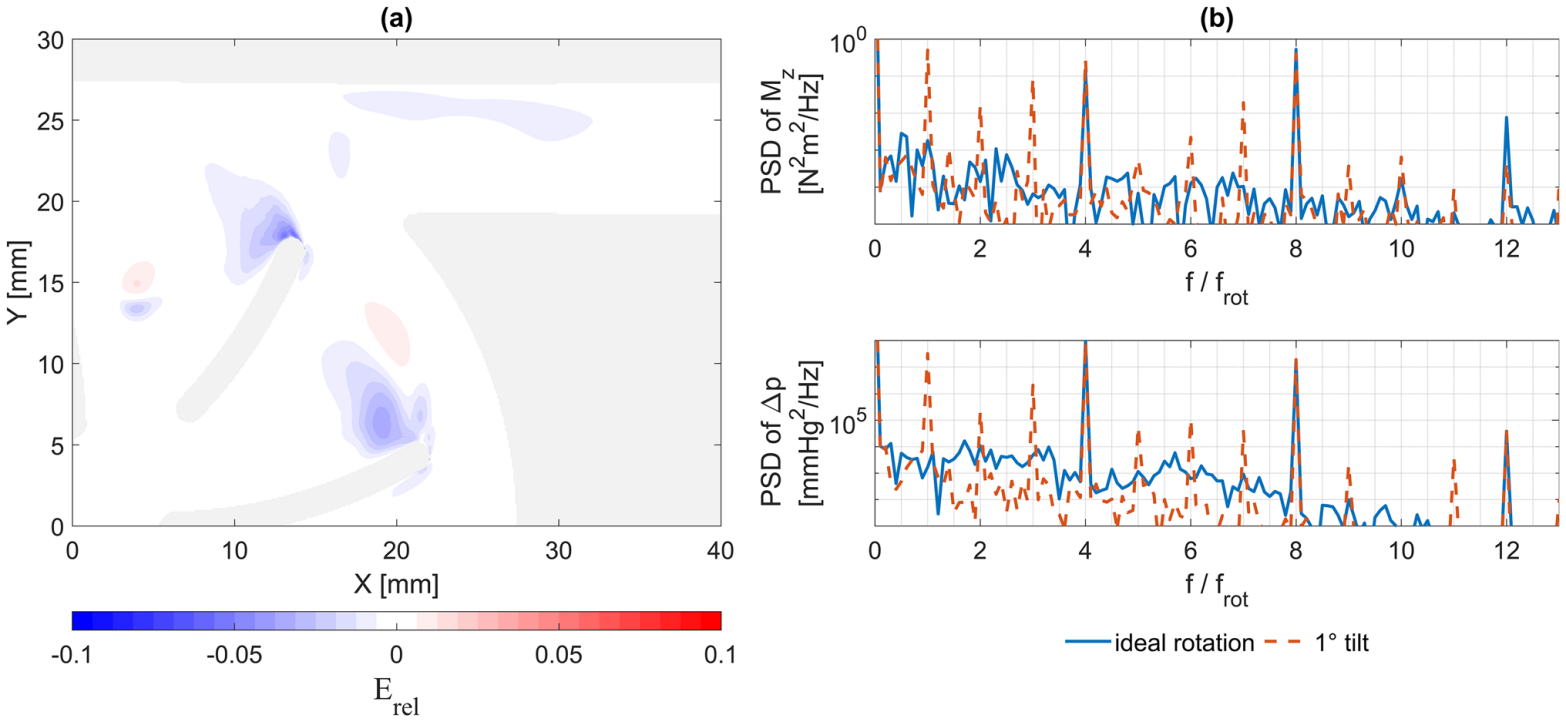
**a** Impact of the simulated non-ideal rotation with the impeller tilted 1° from its vertical rotational axis. The panel shows the relative, normalized error ${\mathrm{E}}_{rel}$ as defined in (3). **b** Power spectral densities (PSD) of the Z-moment on the rotor (${\mathrm{M}}_{{\mathrm{z}}}$) and the pressure head (${\Delta p}$) for the ideal and non-ideal rotation over the frequency spectrum ${\mathrm{f}}$, normalized by the rotational frequency (${\mathrm{f}}_{{{\mathrm{rot}}}}$)

 Figure 6a shows the normalized deviation of the phase-averaged velocity field of the non-ideal rotation from that of the ideal rotation with the impeller at $\alpha = 0^\circ$, being the one with the highest RMS. While a small impact on the velocity field can be observed, primarily in the region of the blade tip wake, it remains highly localized and its magnitude is minute, with an RMS of 0.51%. This deviation is an order of magnitude smaller compared to, e.g., the difference between two RANS models, as shown in Section *Comparison Between LES & RANS*. The other two observed rotor positions at 30° and 60° had RMS errors of 0.41% and 0.49%, respectively.

An interesting observation for future investigations can be made by comparing the power spectral densities of the rotor moment and pressure head (Fig. 6b) for the ideal and non-ideal rotation cases. As expected, we see modes at the passage frequencies of the main blades and secondary blades (at 4 and 8 times the rotational frequency) for all cases. Uniquely, the tilted case exhibits an additional mode at the rotational frequency itself as well as its harmonics at multiples of the rotational frequency.

### PIV Measurements and Comparison with LES

Under the test conditions, the average measured pressure head in the PIV setup was 122.4 mmHg. The average pressure head predicted by LES was 123.9 mmHg over twelve rotations, corresponding to a relative deviation of 1.2% from the experimental measurements.

**Fig. 7:**
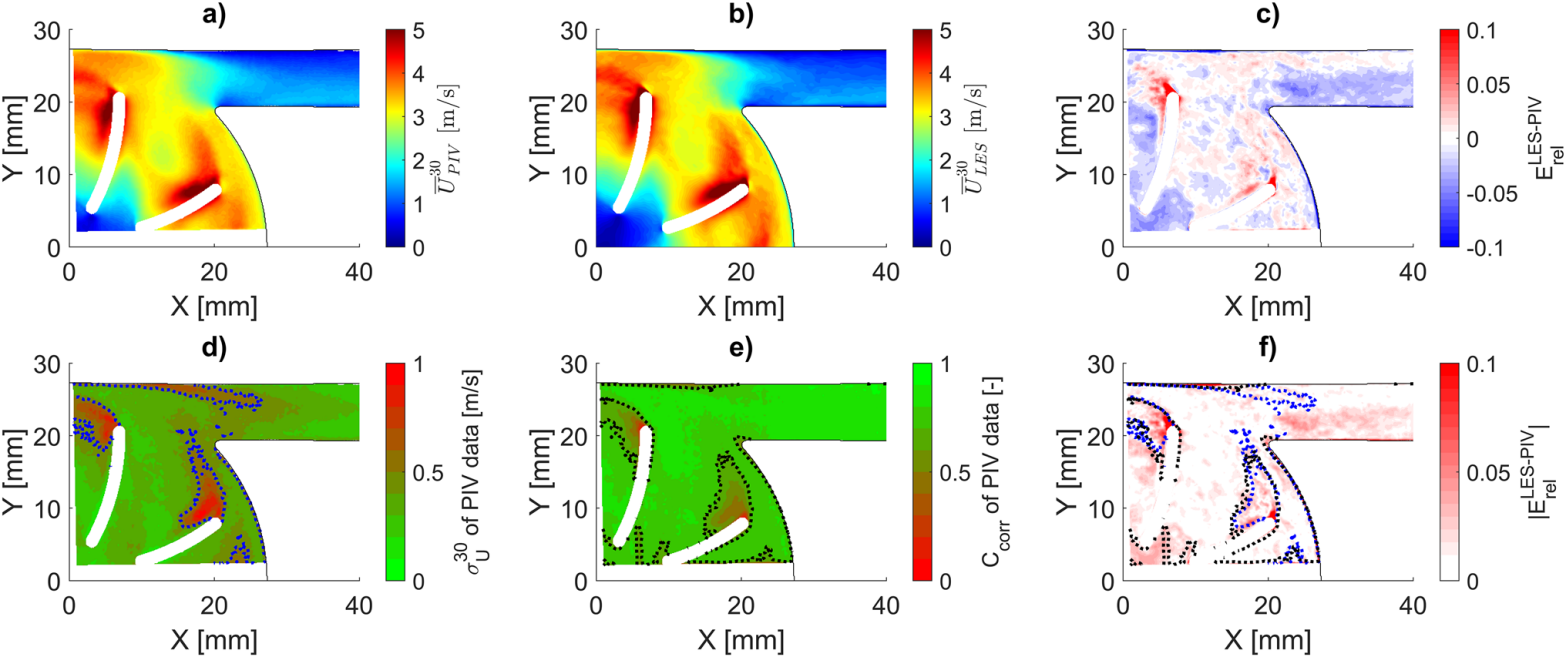
Comparison of the phase-averaged velocity fields for impeller position at 30° $\left( {{\overline{U}}^{30} } \right)$, as measured by PIV and predicted by LES. **a** PIV measurements, phase-averaged over 2.3 s. **b** LES prediction, phase-averaged over 8 rotations. **c** Relative deviation of LES to PIV $\left( {\mathrm{E}_{rel} \left( {\overline{U}^{30} } \right)} \right)$ as defined in (3). **d** Standard deviation of the velocity magnitude $\left( {\sigma_{U}^{30} } \right)$, as defined in (17). **e** Phase-averaged correlation factor of the PIV analysis, ${C}_{corr}$. **f** Relative error magnitude of LES to PIV $\left( {\left| {{\rm E}_{rel} \left( { \overline{U}^{30} } \right)} \right|} \right)$, overlaid with the threshold isolines of ${\upsigma }_{U}^{30} = 0.7$ m/s (blue) and ${C}_{corr} = 0.5$ (black)

 Figure 7 shows the phase-averaged velocity magnitude of the PIV and LES data exemplarily for a main blade angle of 30° as well as the normalized deviation between the two. Further depictions for angles of 0° and 60° are available in the supplementary material (Fig. S6 and S7). For all blade angles, we observed very good agreement of the two velocity fields in terms of general flow pattern and velocity magnitude. The RMS deviation across the investigated field ranges from 2.70% to 2.81% for the different blade angles. The normalized deviation exceeds ± 10% only in very localized areas within the wake of the blades and close to the housing walls.

The larger deviations in the blade wake are likely linked to the highly turbulent, high-speed nature of the flow in this region. Figure 7d shows the standard deviation across all PIV velocity fields ($\sigma_{U}$) for a given phase angle $\alpha$,

17 $$\sigma_{U}^{\alpha } \left( {X,Y} \right) = \frac{1}{{N^{\alpha } }}\mathop \sum \limits_{n = 1}^{{N^{\alpha } }} \left( {U_{n}^{\alpha } \left( {X,Y} \right) - \overline{U}^{\alpha } \left( {X,Y} \right)} \right)^{2}$$

where $X$ and $Y$ indicate the location, $n$ is the numerator of $N^{\alpha }$ available fields for phase angle $\alpha$, and $U$ and $\overline{U}$ are the instantaneous and phase-averaged velocity magnitude with $\overline{U}$ being computed according to (2). $\sigma_{U}$ clearly flags regions of high variation within the blade wake, pointing toward a turbulent flow field. Additionally, Fig. 7e shows that the phase-averaged correlation coefficient ($C_{corr}$, as implemented in PIVlab) drops significantly in the blade wake, pointing toward a less robust tracking of particle clusters in this region.

Overlaying isolines of specific thresholds of both $\sigma_{U}$ and $C_{corr}$ onto the error map, as shown in Fig. 7f, reveals a strong spatial correspondence between these indicators. Supplementary Fig. S8 further displays the time-averaged total turbulent kinetic energy (TKE), as estimated from LES using the formulation described in [20]. The high TKE observed in the blade wake confirms the highly turbulent nature of the flow in this region and aligns well with the areas of low correlation.

Another potential contributor to low correlation is the loss of tracer particles between image pairs due to out-of-plane motion. To assess this, we used the LES-predicted *Z*-velocity component to estimate the particle displacement between two PIV frames acquired at 10,000 fps. The resulting phase-averaged *Z*-displacement fields are shown in supplementary Fig. S8. Although the blade wake exhibits the highest *Z*-velocity, displacements only exceed the commonly accepted threshold of 25% of the laser sheet thickness [24, 25] in very localized areas, and the spatial distribution of strong out-of-plane motion does not align well with the areas that show a low PIV correlation coefficient. This suggests that out-of-plane motion is not a dominant contributor to reduced correlation in the blade wake.

Another region of noticeable deviation, although remaining well below 10%, is the transition from the volute into the outlet cannula. This region has been shown to be instable and hard to predict in other studies of centrifugal VADs, such as the FDA study [9], where it exhibited the largest error compared to the PIV reference data for almost all CFD entries and also the largest spread of results between the different CFD entries.

### Comparison Between LES and RANS

LES and uRANS simulations were carried out on the *Euler* high-performance computing cluster at ETH Zurich (see Table S2 in the supplementary for hardware specifications). Jobs were submitted with *SLURM* requesting 768 cores and 4 GB memory per core for the LES and 256 cores and 2 GB per core for the uRANS. uRANS cases consumed between 1600 and 1900 core-hours per rotor revolution (depending on the turbulence closure model), whereas the LES required approximately 95,000 core-hours per revolution. To exclude time consumed by data output for intermediate results, core-hours were obtained from the CPU-time per time-step reported by the solver and therefore reflect processor time rather than queue time. Although the heterogeneous node architectures mean these core-hour totals are not directly comparable, they still provide an approximate measure of the relative computational cost of uRANS and LES.

The average pressure heads predicted by the uRANS models were 125.3 (k-ω SST), 126.3 (k-ε EB), and 119.4 mmHg (RST). The largest deviation from the LES was observed for the RST model, with −3.6%. The k-ω SST and k-ε EB models were notably closer to the LES prediction, showing deviations of 1.2% and 2%, respectively.

**Fig. 8:**
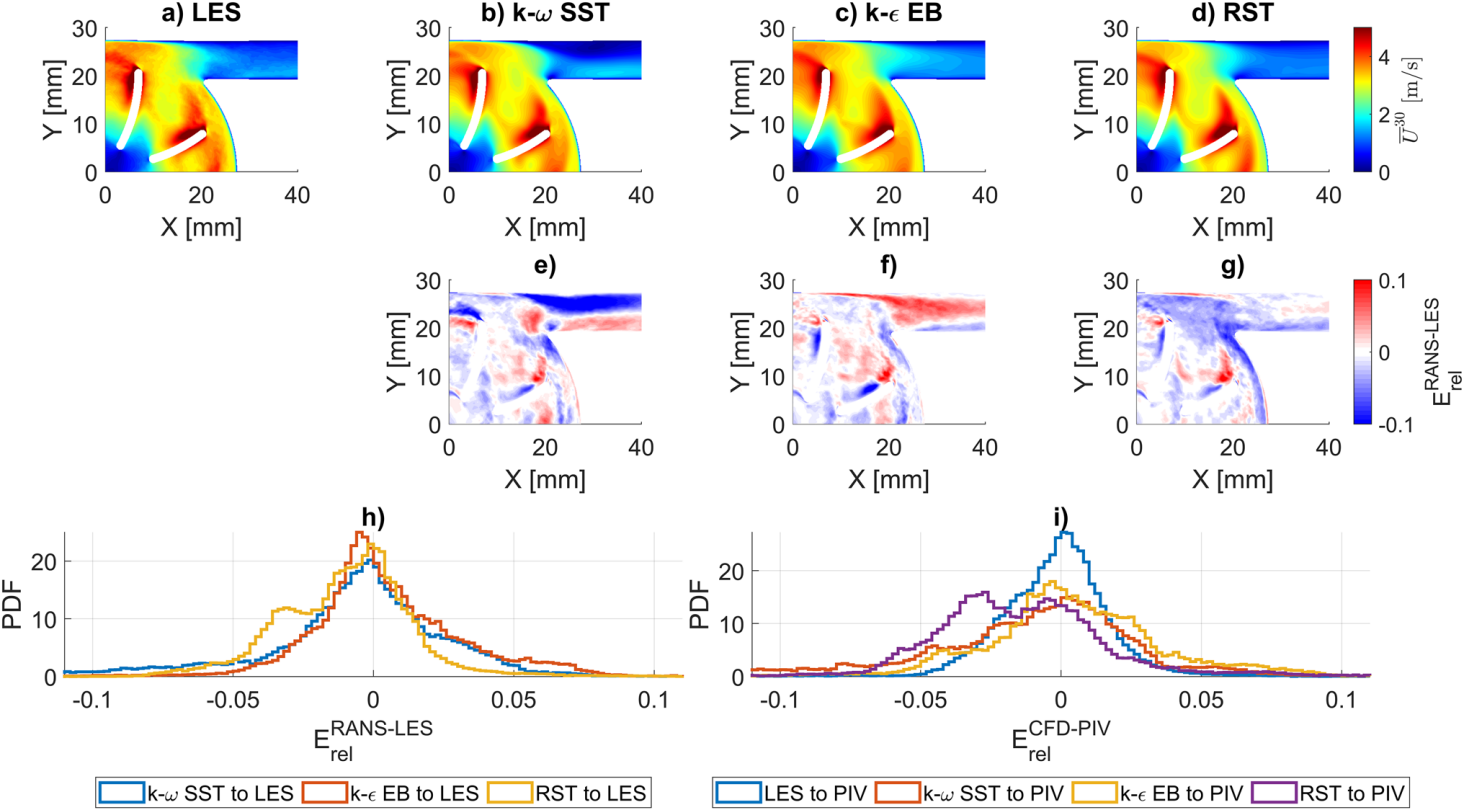
Velocity fields for the LES and RANS and their respective deviations for the impeller position at 30°. **a**–**d** Phase-averaged velocity magnitude fields ${\overline{\mathrm{U}}}^{30}$ of LES, k-ω SST, k-ε EB, and RST (from left to right). **e**–**g** Relative deviation of the ${\overline{\mathrm{U}}}^{30}$ predictions $\left( {{\rm E}_{rel} \left( { \overline{U}^{30} } \right)} \right)$ between the RANS and the LES as defined in (3). **h** PDF of ${\rm E}_{rel} \left( { \overline{U}^{30} } \right)$ between RANS and LES. **i** PDF of $\mathrm{E}_{rel} \left( {\overline{U}^{30} } \right)$ between CFD and PIV

 Figure 8 shows the phase-averaged velocity fields at $\alpha = 30^\circ$ for the LES and all uRANS models as well as the normalized deviation of the uRANS results from the LES. The same analysis for $\alpha = 0^\circ$ and $\alpha = 60^\circ$ are available in the supplementary material (Fig. S9 and S10) as well as a similar comparison for the pressure fields (Fig. S11, S12, and S13).

The most pronounced deviation in the velocity fields is located in the outlet cannula. Especially, the k-ω SST and k-ε EB models misrepresent the orientation of the jet entering the cannula from the volute. The k-ω SST predicts a stronger initial misalignment of the jet relative to the cannula axis, while the k-ε EB deviates in the opposite direction. Within the main volute region, the largest deviations of uRANS from the LES are similar in location and magnitude to the deviation of the LES from PIV. The uRANS RST model shows the closest qualitative match with the LES velocity field and has the smallest RMS error, ranging from 2.48% to 2.57% (depending on blade position). The k-ε EB and k-ω SST RMS are only slightly higher, ranging from 2.75% to 2.81% and 3.93% to 4.14%, respectively.

 Figure 8h shows the probability density function (PDF) of the error distribution of the three different RANS model results with respect to the LES as reference. k-ω SST and k-ε EB show very similar error distributions with a tendency to overpredicting velocity magnitude relative to LES, while RST tendentially underpredicts. We have observed this difference between the Boussinesq approximation-based models and the RST previously [20]. Figure 8i illustrates the error distributions of all CFD model results with respect to the PIV measurements. It is evident that the LES provides the closest match to the experimental data, while the RANS models display a wider spread in the error distribution.

In the pressure fields (depicted in the supplement), the k-ε EB model shows the biggest deviation with an absolute RMS of 3.31 to 4.29 mmHg, caused by a general overprediction with a mean pressure deviation of 2.29 to 3.44 mmHg. The RST model performs better with an RMS of 2.53 to 2.70 mmHg, but its error distribution shows two peaks, one around 0 mmHg and a second around −4 mmHg. The second peak is caused by an underprediction of the pressure increase from the volute to the outlet cannula, leading to a negative pressure offset within the entire outlet cannula when compared to the LES. The k-ω SST performs best in terms of pressure prediction with the smallest mean error of −0.18 to 0.14 mmHg and an RMS of 2.02 to 2.31 mmHg.

## Discussion

In this study, we embarked on a comprehensive investigation into the suitability of large eddy simulation (LES) as an alternative to experimental flow field quantifications in the validation of RANS models of a magnetically levitated centrifugal blood pump. To this end, we conducted PIV measurements of the CentriMag and compared the experimental results with simulated fields obtained from LES and three different RANS models. Our data show that the deviations between LES and PIV fall within the expected accuracy of our PIV setup and are smaller than the inter-laboratory variability of PIV measurements reported in [8]. Within the scope of our study, LES demonstrated the potential to serve as a robust reference for validating RANS models when applied using best practices. To assess the validity of commonly assumed idealized rotor motion in CFD models, we designed our experiments to also capture the actual impeller motion. Our findings suggest that the non-rotatory components of the impeller motion had minimal influence on the resulting flow fields within the observed range of deviations between measurements and simulations, which may support the continued use of idealized rotation in similar computational models.

Comparison of the PIV and LES phase-averaged velocity fields shows a very good agreement between the two modalities, with an RMS error between 2.70% and 2.81% depending on the blade angle. The largest deviations are located in the wake of the blades and along the housing walls. Deviations near the walls are most probably imparted by experimental limitations in that area, including optical interferences with the housing and coarser near-wall resolution than is possible with LES where the height of the first prism layer of 40 µm is smaller than the particle size used in the PIV measurements. In the wake of the blades, the LES predicted higher velocity magnitudes than were measured with PIV, which may be due to an overprediction of the velocity magnitude in the LES or a bias toward lower velocities in the PIV data. The blade wakes are the regions with the highest turbulent kinetic energy. Based on the LES simulations, the Taylor Microscale is estimated to be smaller than 300 µm within the wake, which is smaller than the final interrogation window of the PIV algorithm $\left( {8 px \cdot 60\frac{\mu m}{{px}} = 480 \mu m} \right)$. This suggests that a relevant part of the turbulent motion in the wakes cannot be resolved by our PIV, a hypothesis that is also corroborated by the low PIV correlation factor in this region. To explore whether out-of-plane particle motion may also have played a role, we further evaluated the Z-velocity components predicted by LES. Although the highest out-of-plane particle displacements occurred in the blade wake, they only exceeded the commonly accepted threshold of 25% of the laser sheet thickness in very localized areas. Furthermore, the spatial distribution of this motion did not align well with regions of low correlation. This indicates that out-of-plane motion likely plays a secondary role in the observed low correlation in the blade wakes, with high turbulence being the dominant factor. Overall, these findings suggest that the deviations in the blade wakes are more likely attributable to experimental limitations than to inaccuracies in the LES predictions.

The other region of noticeable deviation at the transition from the volute to the outlet cannula shows neither significant turbulence nor a diminished correlation factor of the PIV data. Other studies investigating flow in centrifugal blood pumps identified the entry into the outlet as being sensitive to operating and boundary conditions [8, 9, 26, 27]. In the FDA benchmark pump, the jet into the outlet was observed to completely switch direction at different RPM, and the outlet region of that pump showed both the highest inter-laboratory variability for PIV measurements [8] and the largest error across all entries of the successive inter-laboratory CFD study [9]. Given the instability of the flow in this region and its sensitivity to boundary conditions, it can be argued that an LES would provide a more reliable reference for RANS validation here, as it allows full consistency of boundary conditions, which an experimental setup cannot provide. Our results confirm the high sensitivity of the volute-to-outlet transition in RANS modeling, consistent with observations in other centrifugal VADs [8, 9, 26, 27], and highlight this region as a particularly suitable target for focused comparisons between different RANS approaches. If experimental validation is to be conducted, regions prone to flow separation, such as the volute-to-outlet transition, should always be included in data acquisition and analysis.

Finally, comparing the LES to the three different RANS predictions, our results show deviations in the phase-averaged velocity fields of a similar magnitude as between LES and PIV with the RST model being slightly more accurate in terms of maximal and RMS errors than the k-ω SST and k-ε EB models. The latter two models also show distinct deviations in flow fields in the region transitioning from the volute to the outlet cannula.

Hariharan et al. compared the independent entries of three laboratories for PIV measurements on the FDA benchmark pump [8]. They state an error of 10% to 30% for the measured pressure head between laboratories and a mean coefficient of variation of the measured velocity field being around 10% in the blade passage region and exceeding 35% in the diffuser region of the pump. Their results clearly show that experimental measurements do not necessarily provide the perfect reference as is often assumed for CFD model validation. Based on this insight and our results, we suggest that LES, if executed with proper care, is a valid alternative reference to PIV measurements for RANS model validation.

Using LES as a reference, either in place or alongside experimental measurements, offers the added benefit of enabling direct comparisons not only of velocity and pressure fields, but also of fluid stress distributions. Accurate prediction of fluid stresses is crucial for assessing the blood damage potential in blood pumps, but is challenging in RANS models due to their inherent limitation in the resolution of turbulence. As demonstrated in our previous work [20], LES can serve as a valuable benchmark for evaluating the accuracy of RANS in predicting stress fields and for comparing methods to incorporate unresolved turbulent stresses in RANS-based models.

Our measurements of the rotor motion showed three modes of motion that deviate from the ideal rotation, including a shift of the rotational center from the global origin, a tilt of the rotation axis in respect to $\mathop{Z}\limits^{\rightharpoonup}$ and a precession of the rotation axis around its mean. Together, these modes of motion lead to the blade tips traveling across a $Z$-range of almost 0.7 mm instead of being level throughout the rotation. Whether these modes can be expected to occur in the real pump remains to be clarified as they may also result, in part or in full, from artifacts of our test assembly. The shift of the rotational center could be caused by the multi-component design of the CentriMag, manufacturing inaccuracies, a spatial calibration error or a combination of all three. Similarly, while the apparent tilt of the mean rotational axis compared to the global $\mathop{Z}\limits^{\rightharpoonup}$-axis of the pump could be explained by inhomogeneous loading of the impeller blades, it could also be explained by an inaccurate alignment of the laser sheet with the $\left( {\mathop{X}\limits^{\rightharpoonup} , \mathop{Y}\limits^{\rightharpoonup} } \right)$ plane. The latter can be ruled out with high certainty as the mean tilt of 0.65° would result in a misalignment in $Z$ of $\approx$ 1.7 mm over the full width of the housing. Given the tolerances of our alignment method using the grooves on the opposing faces of the housing we instead estimate the tolerance of our alignment to lie within 0.25 mm of misalignment in $Z$ over the width of the housing which translates to < 0.1 deg.

Finally, the precession is most likely caused by either a tilted mounting of the magnet within the impeller or a non-uniform magnetization of the magnet, as both would lead to a tilt of the impeller body relative to the electromagnetic field of the drivetrain. Based on our study alone, we can therefore not conclusively state that the measured non-ideal motion should be expected in a standard CentriMag unit. However, we can say with high confidence that our observations provide an upper limit of the expectable deviations from an ideal rotation.

To assess the impact of such departure from an ideal rotation on the pump internal flow fields, we modelled a 1° precession of the rotational axis around the $\mathop{Z}\limits^{\rightharpoonup}$-axis. The blade tips traveled a $Z$-range of 0.77 mm, 10% more than the maximal deviations measured in our experiments. Even under these conditions, the impact on the flow field was minute with an RMS error of the phase-averaged velocity magnitude at the height of the rotor blades ranging between 0.41% and 0.51% for the three investigated angles. Altogether, these results suggest that, for the CentriMag at least, the assumption of an ideal rotation is a valid simplification. We also illustrated that departure from an ideal rotation leads to the emergence of peaks in the power spectrum of the pressure head and rotor moment at frequencies other than the blade passage frequency and its harmonics. Based on a similar phenomenon, Kaufmann et al. used acoustic spectral analysis to detect in-pump thrombosis in the HVAD pump [28]. They showed that thrombus formation in the pump led to peaks in the acoustic spectrum at frequencies different to the normally present harmonics of the rotary frequency which they linked to eccentric motion of the rotor. We suggest that analysis of the power spectra of integral measures such as the pressure head or rotor moment can serve to identify non-rotatory motion modes without having to characterize blade motion in detail.

Our study has the limitation that it was exclusively performed on the CentriMag and under a single operating condition. However, the results presented here can be assumed to be translatable to other operating points and rotatory blood pumps at least in a qualitative manner. To evaluate the transferability of our results on flow modeling to other pumps, several factors should be considered carefully, such as the employed grid setup and numerical methods as well as the present flow regime and boundary conditions. We specifically chose a wall-resolved LES setup as it does not limit the applicability of our results to a specific wall-layer model which are known to be problem dependent in their accuracy [29, 30]. Consequently, this limits our insights on the validity of LES to setups with high enough grid resolution to achieve the $y^{ + }$ and CFL numbers necessary for wall-resolved LES. The employed WALE sub-grid scale model still contains an adjustable, empirical model coefficient (as do all LES models), which is not universal [29]. The WALE model is, however, less sensitive to the value of its model coefficient than the original Smagorinsky model and has shown superior accuracy over other models for a wide range of applications [31–35]. The similarity of flow regimes between different pumps can be judged by comparing the Reynolds number $Re$, which is generally defined as

18 $$Re = \frac{\rho uL}{\mu }$$

where $u$ is the velocity, $L$ is a representative length scale, and $\mu$ is the dynamic viscosity. For pipes such as the pump inlet, the inner diameter of the inlet $D_{inlet}$ is used as a representative length scale. For rotary pumps, the formulation can be adapted to the pump Reynolds number:

19 $${Re}_{pump} = \frac{{\rho \dot{\omega }D_{rotor}^{2} }}{\mu }$$

where $\dot{\omega }$ is the angular velocity of the rotor in rad/s and $D_{rotor}$ is the rotor diameter.

Table 1 lists the ranges of $Re_{inlet}$ and $Re_{pump}$ for the CentriMag, the HeartMate 3, and the FDA benchmark pump. While the absolute numbers differ by factors of roughly 0.5−2 from the investigated condition, $Re_{pump}$ for all pumps lies well within the turbulent regime, while $Re_{inlet}$ in all pumps represents transitional flow. Despite the varying designs of these pumps, they exhibit similar flow regimes, a wide range of which is covered by the investigated operating condition in the CentriMag. Combined with the generally similar design and flow path of these pumps, this indicates that the insights gained in this study are transferable to other currently relevant centrifugal blood pumps. The main limitation could be pumps in which a true transition from fully laminar to turbulent flow occurs, a phenomenon which our study does not cover but which is also not likely to occur in this environment. On the other end of the spectrum, exceedingly high $Re$ might present a practical limit, as higher $Re$ require successively higher grid resolution.

**Table 1:** Reynolds regimes of the CentriMag, the HeartMate 3, and the FDA benchmark pump at different operating points

	${\Delta p}$ (mmHg)	${\mathrm{Q}}$ (L/min)	${\dot{\omega }}$ (rpm)	${\mathrm{D}}_{{{\mathrm{inlet}}}}$ (mm)	${\mathrm{D}}_{{{\mathrm{rotor}}}}$ (mm)	${Re}_{\mathrm{inlet}}$ (-)	${Re}_{\mathrm{pump}}$ (-)
CentriMag, tested VAD operation	110	4.4	2350	9.2	44.9	2804	137,100
CentriMag, ECMO operation [16]	350	5	4050	9.2	44.9	3187	236,300
HeartMate 3, partial support [35]	80	2.5	5000	7.1	18.7	2065	50,590
HeartMate 3, full support [36]	80	5	5650	7.1	18.7	4129	57,170
FDA benchmark pump, FDA condition #1 [9, 12]	170	2.5	2500	12	52	1222	195,600
FDA benchmark pump, FDA condition #5 [9, 12]	260	6	3500	12	52	2932	273,800

To evaluate the transferability of our results on non-rotatory motion, we can consider possible stabilizing and destabilizing factors in the CentriMag and weigh them against those in other pumps. Compared to other MagLev VADs, such as the HeartMate 3, the CentriMag has an unusually large rotor diameter and blade size relative to its magnet diameter. As a result, possibly destabilizing hydrodynamic forces on the CentriMag’s rotor are likely to be more critical relative to stabilizing moments of the magnetic drivetrain. In a first approximation, it can, therefore, be expected that other MagLev VADs will present a lower magnitude of non-rotatory motion, which, according to our results, will have a negligible impact on the flow. Our suggested approach of analyzing power spectra of integral measurements additionally provides an easy-to-implement method to check for non-ideal rotation.

## Conclusion

In light of the recent focus of the FDA on the use of CFD in medical device development, several studies have addressed the need for rigorous validation of CFD models [7]–[10, 37]. Experimental validation, whether through integral measures such as pressure head and flow rate or through more detailed velocity field measurements, is often regarded as the only reliable approach. Our findings challenge this assumption in the context of RANS model validation for blood pumps by demonstrating that, in this study, the discrepancies between PIV measurements and LES predictions fall within the expected accuracy range of the PIV itself. Within the scope of our investigation, LES can be considered a viable alternative to experimental flow field quantifications for validating RANS models of blood pumps. If experimental validation is to be pursued in a limited setup, our results suggest that the volute-to-outlet transition region is a logical starting point. This area is known to exhibit unstable flow behavior and has proven challenging to predict accurately in previous studies across various pumps and setups [8, 9, 27, 36]. Furthermore, its accessibility makes it well suited for optical measurement techniques. It may thus serve as a particularly sensitive region to obtain an initial indication of model fidelity.

We also showed that a magnetically levitated impeller in a modern centrifugal blood pump can exhibit small but complex non-rotary components. However, in our setup, these deviations had negligible impact on the flow field. Since the assumption of idealized rotation had not been previously verified, our results provide the first experimental confirmation that this simplification is appropriate, at least within the bounds examined here.

## Supplementary Information

Below is the link to the electronic supplementary material.

Supplementary file1 (PDF 2413 kb)
