# Supplementary material for: Experimental Validation of Large Eddy Simulation as a Benchmark for Reynolds-Averaged Navier-Stokes Flow Modeling in a Magnetically Levitated Blood Pump
Source: Ann Biomed Eng. 2025 Dec 9;54(3):719–36. doi: 10.1007/s10439-025-03846-4 (PMC12960318; doi:10.1007/s10439-025-03846-4)
Supplement: Supplementary file 1 — Supplementary file1 (PDF 2413 kb) [file 10439_2025_3846_MOESM1_ESM.pdf]

Annals of Biomedical Engineering

Supplementary Material

Experimental Validation of Large Eddy Simulation as a Benchmark for Reynolds-Averaged Navier Stokes Flow Modelling in a Magnetically Levitated Blood Pump

Jonas Abeken[1], Utku Gülan[2], Kai von Petersdorff-Campen[3], Vasileios Charitatos[1], Marianne Schmid-Daners[4], Mirko Meboldt[3], Markus Holzner[5], Diane de Zélicourt[1], Vartan Kurtcuoglu[1]

[1] Interface Group, Department of Physiology, University of Zurich, Switzerland
[2] Hi-D Imaging, Winterthur, Switzerland
[3] Product Development Group Zurich, Institute of Materials, Design and Fabrication, ETH Zurich, Switzerland
[4] Institute for Dynamic Systems and Control, ETH Zurich, Switzerland
[5] Environmental Fluid Mechanics Group, Institute of Hydraulic Engineering and River Research, University of Natural Resources and Life Sciences BOKU, Vienna, Austria

Corresponding Author: Vartan Kurtcuoglu, vartan.kurtcuoglu@uzh.ch

I. Supplementary Methods

A. Complete List of PIVlab Settings

Table S1 lists the complete set of parameters used in the PIVlab toolbox in Matlab to generate the velocity fields from the experimental footage.

Table S1: Detailed settings used in the PIVlab Matlab script

**Image Preprocessing**	
CLAHE	enabled (window size: 64 pixels)
Highpass	disabled
Intensity Capping	disabled
Wiener2 Denoise	disabled
Contrast Stretch	Auto (min.: 0; max.: 1)
Background Subtraction:	Enabled
**PIV Settings**	
PIV algorithm	FFT window deformation
Pass 1 interrogation area	64
Pass 2 interrogation area	32
Pass 3 interrogation area	16
Pass 4 interrogation area	8
Repeat last pass until quality slope	0.025
Sub-pixel estimator	Gauss 2x3-point
Correlation robustness	Extreme
**Image based validation**	
Filter Low contrast	Enabled (Threshold: 0.002)
Filter bright objects	Enabled (Threshold: 0.9)
Correlation coefficient filter	Enabled (Threshold: 0.5)
Interpolate missing data	Disabled
**Velocity based validation**	
Velocity limits	Disabled
Standard Deviation Filter	Disabled
Local median filter	Enabled (Threshold: 3)
Magnitude notch filter	Disabled
Interpolate Missing Data	Disabled

B. PIV Housing Calibration Grids

Fig. S1 depicts the bottom part of the PIV housing replica with the implemented grooves for alignment of the laser sheet and the two grids for spatial calibration of the footage.

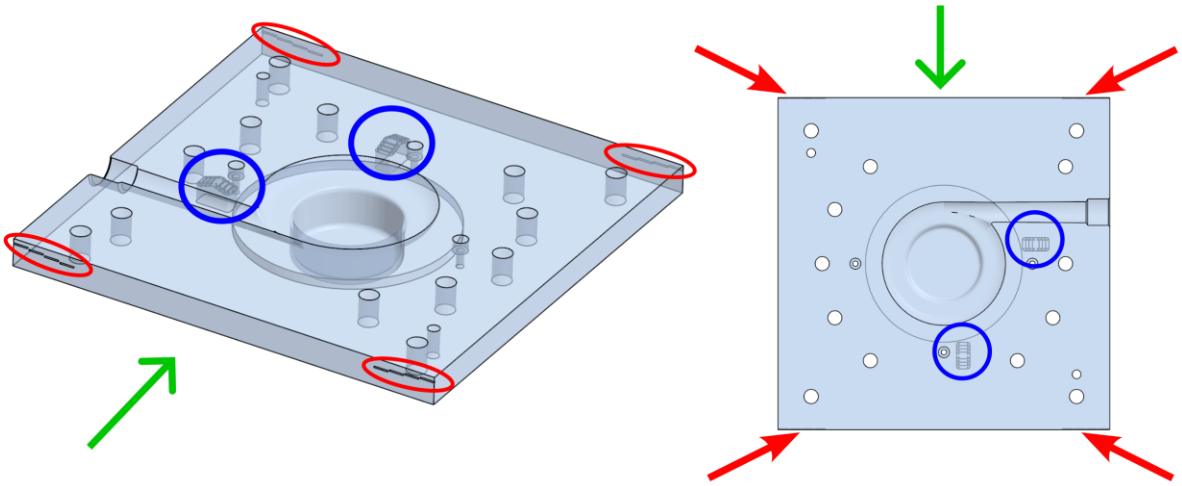

Fig. S1: *Isometric (left) and top-view (right) of the bottom part of the PIV housing replica showing the side grooves for alignment of the laser sheet (red ovals and arrows) and the calibration grids (blue circles). The green arrows indicate the direction of the laser sheet.*

C. Optical Measurement of Impeller Position

1. Simplification of $z_n^{in} \approx z_n^{out} = z_n$

As derived in the manuscript, we can express the distance between centre points of the illuminated markers of a main blade d_n as a function of the heights z_n^{in} and z_n^{out} at which the laser plane intersects the inner and outer channels:

$$d_n(z_n^{in}, z_n^{out}) = \sqrt{(x_n^{out}(z_n^{out}) - x_n^{in}(z_n^{in}))^2 + (y_n^{out}(z_n^{out}) - y_n^{in}(z_n^{in}))^2 + (z_n^{out} - z_n^{in})^2} \tag{S-1}$$

The angulation of the segment $I_n^{in} I_n^{out}$ relative to the impeller reference plane varies depending on the blade location but is at most equal to the impeller's tilt angle θ. The height difference between I_n^{in} and I_n^{out} from the rotor plane is thus bounded by

$$0 \leq \left|z_n^{out} - z_n^{in}\right| \leq d_n \cdot \sin(\theta) \tag{S-2}$$

The tilt of the impeller is limited to $\theta < 2.5°$ as the impeller would collide with the housing at higher tilt angles. Given this, the height difference can be assumed to be approximately zero relative to the other distances in (S-1), i.e. $\left|z_n^{out} - z_n^{in}\right| \approx 0$.

The same argument can be used to simplify

$$P_n = \begin{pmatrix} X_{I_n^{in}} \\ Y_{I_n^{in}} \\ Z_{I_n^{in}} \end{pmatrix} - R_{\vec{z}}(\alpha) \cdot R_{\vec{\psi}}(\theta) \cdot \begin{pmatrix} a_n^{in} \cdot z_n \\ b_n^{in} \cdot z_n \\ z_n \end{pmatrix}. \tag{S-3}$$

The rotation matrix $R_{\vec{\psi}}(\theta)$ around an arbitrary vector $\vec{\psi} = (\psi_X, \psi_Y, \psi_Z)$ by θ is defined as

$$R_{\vec{\psi}}(\theta) = \begin{bmatrix} \psi_X^2(1-\cos(\theta)) + \cos(\theta) & \psi_X\psi_Y(1-\cos(\theta)) - \psi_Z\sin(\theta) & \psi_X\psi_Z(1-\cos(\theta)) + \psi_Y\sin(\theta) \\ \psi_X\psi_Y(1-\cos(\theta)) + \psi_Z\sin(\theta) & \psi_Y^2(1-\cos(\theta)) + \cos(\theta) & \psi_Y\psi_Z(1-\cos(\theta)) - \psi_Z\sin(\theta) \\ \psi_X\psi_Z(1-\cos(\theta)) - \psi_Y\sin(\theta) & \psi_Y\psi_Z(1-\cos(\theta)) + \psi_Z\sin(\theta) & \psi_Z^2(1-\cos(\theta)) + \cos(\theta) \end{bmatrix}. \tag{S-4}$$

With $\theta < 2.5°$ we can assume $\cos(\theta) \approx 1$ and $\sin(\theta) \approx 0$ which simplifies above equation to the identity matrix

$$R_{\overline{\psi}}(\theta) = \begin{bmatrix} 1 & 0 & 0 \\ 0 & 1 & 0 \\ 0 & 0 & 1 \end{bmatrix}, \tag{S-5}$$

allowing the simplification of (S-3) to

$$P_n \approx \begin{pmatrix} X_{I_n^{in}} \\ Y_{I_n^{in}} \\ Z_{I_n^{in}} \end{pmatrix} - R_{\vec{z}}(\alpha) \cdot \begin{pmatrix} a_n^{in} \cdot z_n \\ b_n^{in} \cdot z_n \\ z_n \end{pmatrix}. \tag{S-6}$$

2. Camera view on the impeller with fluorescent blade marker channels

Fig. S2a shows the top view corresponding to the side view schematic of Fig. 3 in the manuscript. Fig. S2b shows the corresponding camera view with the illuminated blade markers clearly visible.

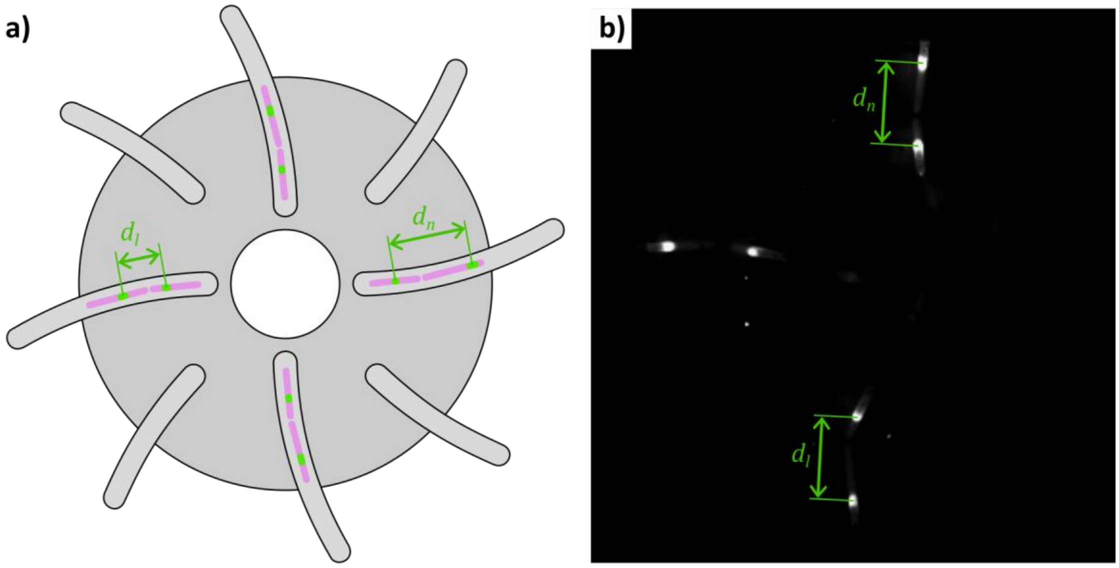

Fig. S2 *a) Schematic of the impeller with the marker channels depicted in pink and the intersection of marker channels and laser-sheet in green with the view axis aligned with $\vec{z}$. b) Extract of a recorded frame showing the marker-pairs of three main blades as bright white ovals. The full marker channels are visible in a faint grey. The depicted image has already been processed to remove background. Before extracting the coordinates of the illuminated markers, an additional step of thresholding the image into binary black & white would be performed.*

3. Hardware specifications of the *Euler* high-performance computing cluster

The *Euler* high-performance computing cluster at ETH Zurich contains CPU nodes of differing architectures. The hardware specifications of the nodes operational at the time of this study are listed in Table S2.

Table S2: Hardware specifications of the Euler high-performance computing cluster.

Node type	Number of Nodes	Sockets / Node	Cores / Socket	CPU model	Base GHz	RAM / Node (GB)	Network
Type 1	216	2	64	AMD EPYC 7742	2.25	512	100 Gb/s InfiniBand HDR
Type 2	292	2	64	AMD EPYC 7H12	2.60	256	100 Gb/s InfiniBand HDR
Type 3	248	2	64	AMD EPYC 7763	2.45	256	100 Gb/s InfiniBand HDR
Type 4	192	2	64	AMD EPYC 7742	2.25	512	100 Gb/s Ethernet
Type 5	192	2	96	AMD EPYC 9654	2.40	384	100 Gb/s InfiniBand HDR

II. Supplementary Results

A. Geometrical Accuracy of CentriMag Replica

Fig. S3 shows the deviation of the scanned surfaces of the milled PMMA replicas from the CAD models of housing halves and the rotor, computed along the normal of the CAD surface.

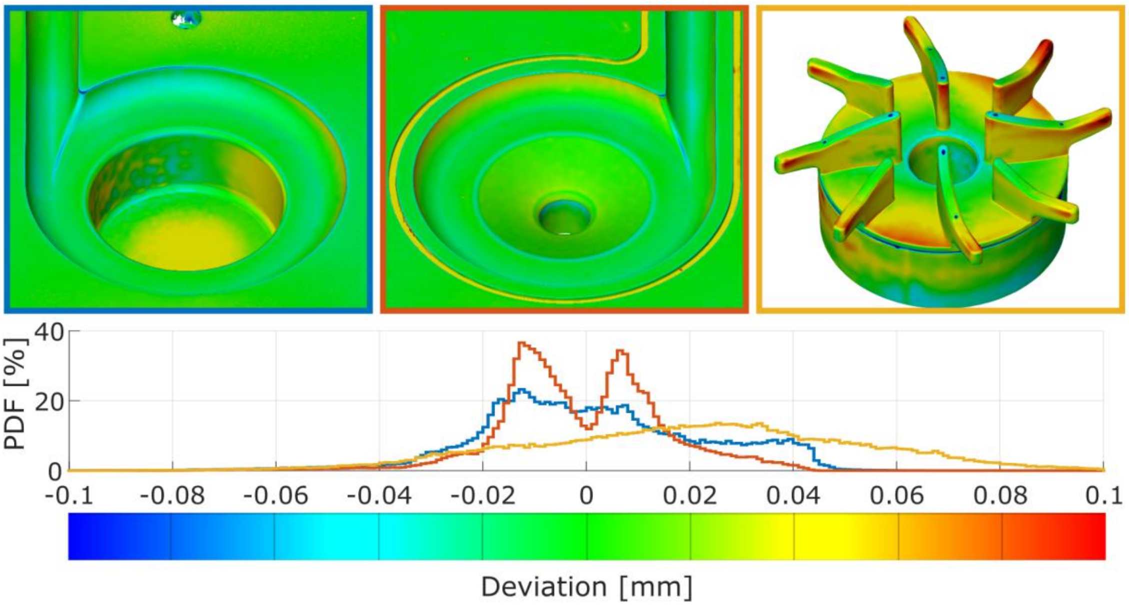

Fig. S3 *Deviation in normal direction of the scanned surfaces of the manufactured replicas of the housing and rotor used for the PIV experiments from the respective CAD models. The graphs show the histograms of the deviations of fluid-contacting surfaces of rotor (yellow line), housing top (red), and housing bottom (blue).*

B. CFD Investigation of Non-Ideal Rotation

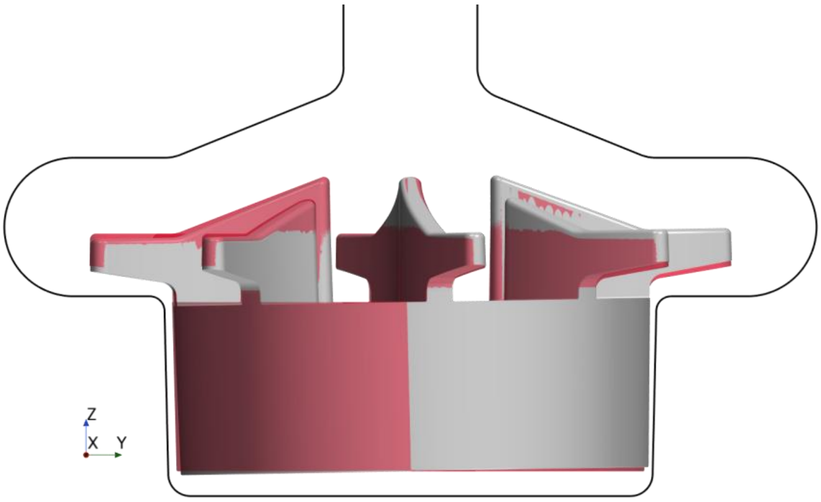

Fig. S4: *Position of the 1° tilted impeller (grey) overlayed with the non-tilted position (red, transparent). The section through the housing surface is shown as a black outline.*

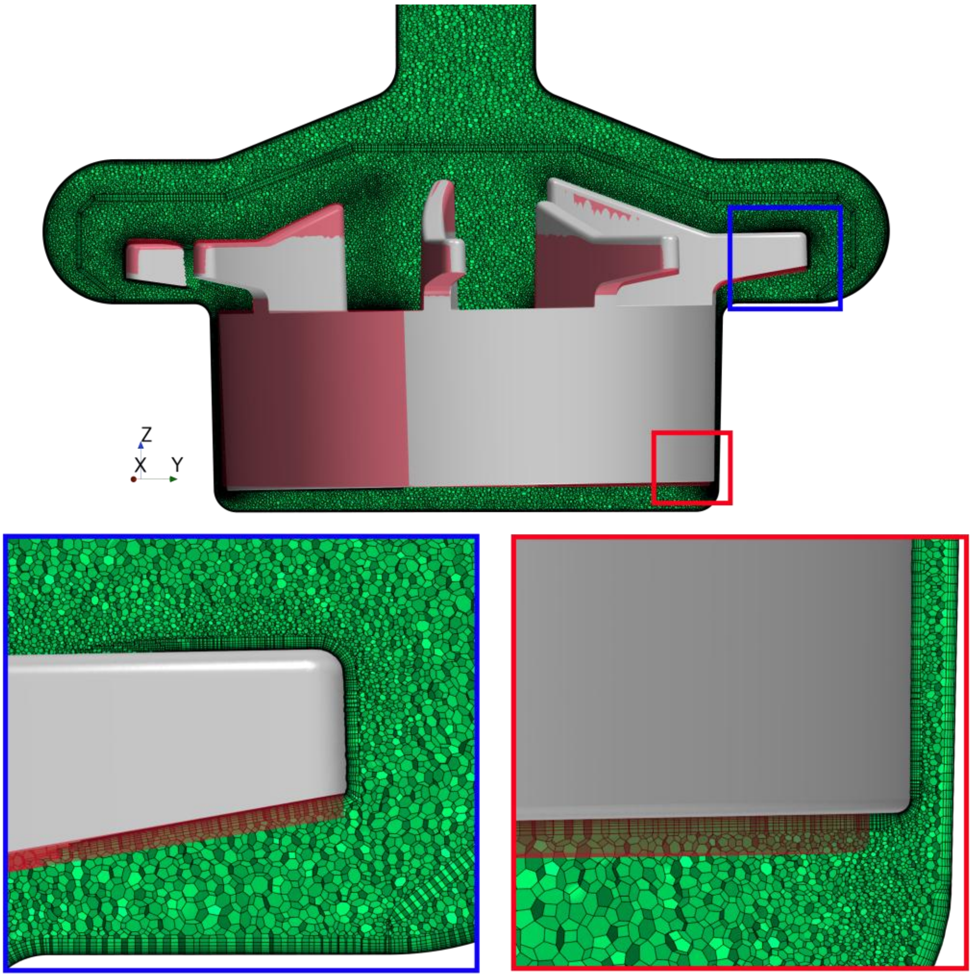

Fig. S5: *Overview and close-up views of the mesh used for the tilted rotor investigation. The non-tilted position of the impeller is shown with the red, transparent overlay.*

C. Comparison Between LES & PIV

Fig. S6 and Fig. S7 show the comparison and analysis of PIV and LES results as shown and discussed in the manuscript for additional main-blade angles of 0° and 60°, respectively.

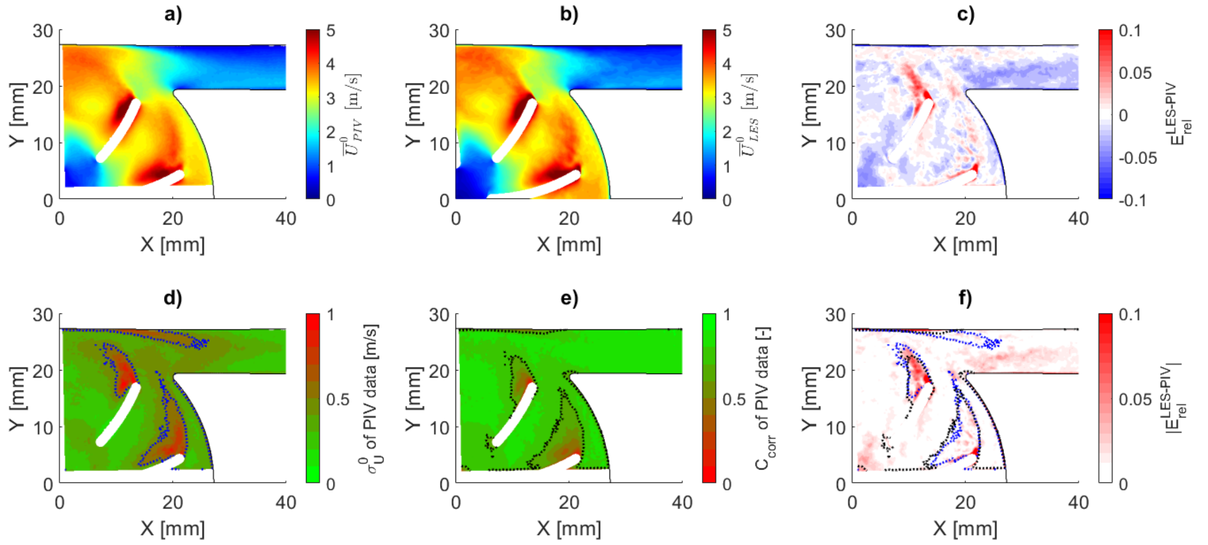

Fig. S6 *Comparison of the phase-averaged velocity fields for impeller position at 0° ($\overline{U}^{0}$), as measured by PIV and predicted by LES. a) PIV measurements, phase-averaged over 2.3s. b) LES prediction, phase-averaged over 12 rotations. c) Relative deviation of LES to PIV ($E_{rel}(\overline{U}^{0})$) as defined in the manuscript. d) Standard deviation of the velocity magnitude (σ_u^0), as defined in the manuscript. e) Phase-averaged correlation factor of the PIV analysis, C_{corr}. f) Relative error magnitude of LES to PIV ($|E_{rel}(\overline{U}^{0})|$), overlayed with the threshold isolines of $\sigma_u^0 = 0.7$ m/s (blue) and $C_{corr} = 0.5$ (black).*

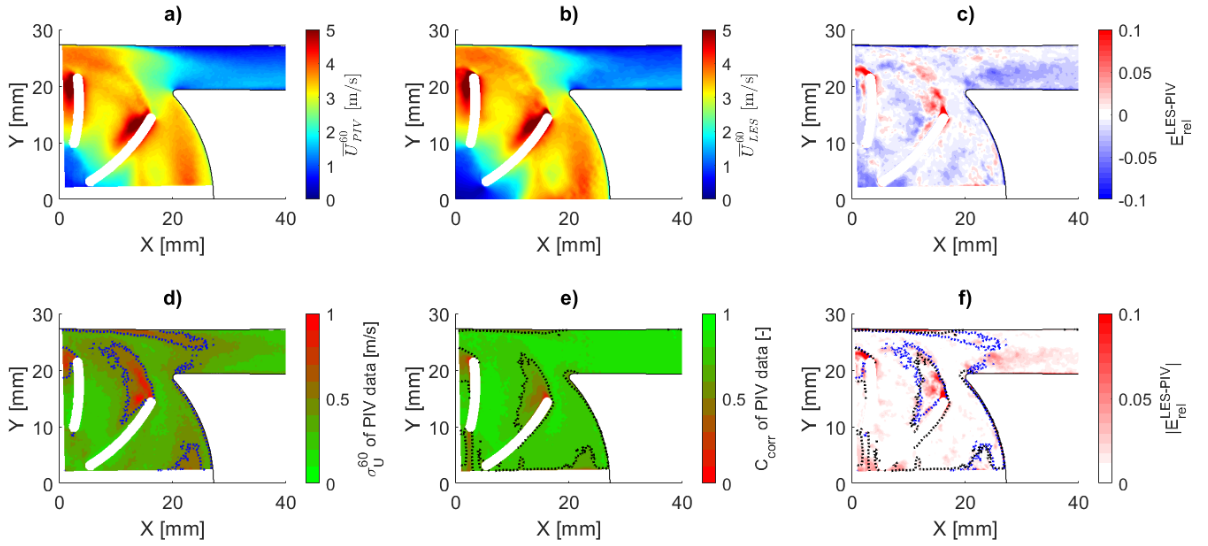

Fig. S7 *Comparison of the phase-averaged velocity fields for impeller position at 60° ($\overline{U}^{60}$), as measured by PIV and predicted by LES. a) PIV measurements, phase-averaged over 2.3s. b) LES prediction, phase-averaged over 12 rotations. c) Relative deviation of LES to PIV ($E_{rel}(\overline{U}^{60})$) as defined in the manuscript. d) Standard deviation of the velocity magnitude (σ_u^{60}), as defined in the manuscript. e) Phase-averaged correlation factor of the PIV analysis, C_{corr}. f) Relative error magnitude of LES to PIV ($|E_{rel}(\overline{U}^{60})|$), overlayed with the threshold isolines of $\sigma_u^{60} = 0.7$ m/s (blue) and $C_{corr} = 0.5$ (black).*

Fig. S8 shows the TKE and magnitude of estimated, relative Z-travel of particles between consecutive PIV images and compare their distribution to the correlation factor of the PIV data. The relative Z-travel $Z_{travel,rel}$ of the particles is estimated based on the phase-averaged Z velocity magnitude $\overline{|u_Z|}$ from the LES, the frame rate per second (FPS) of the PIV camera and normalized by the laser sheet thickness $\Delta Z_{lasersheet}$:

$$Z_{travel,rel} = \frac{\overline{|u_Z|}}{FPS \cdot \Delta Z_{lasersheet}}$$

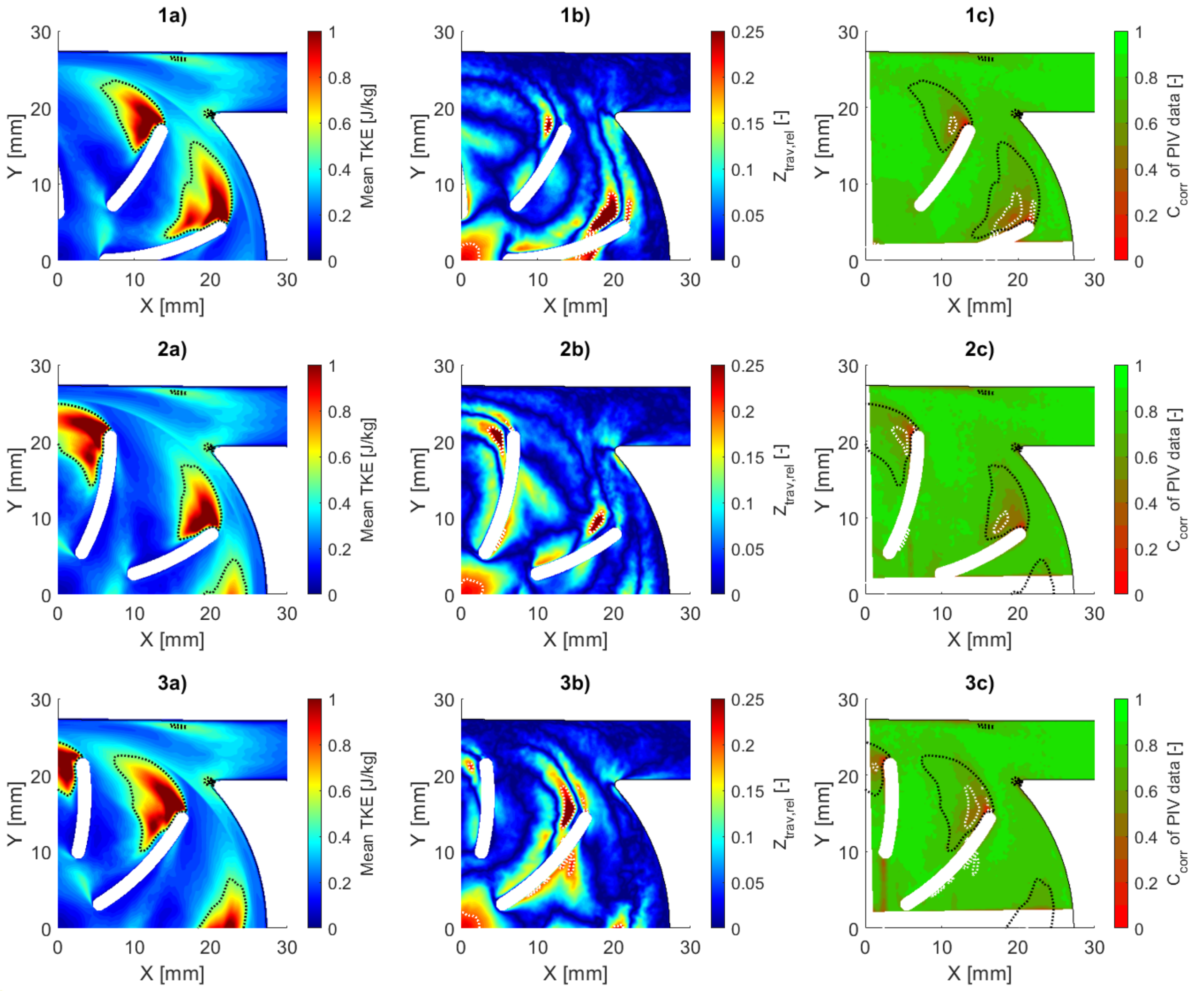

__Fig. S8__ Comparison of the distribution of TKE and Z-travel to the correlation coefficient of the PIV data for impeller position at 0° (1a-c), 30° (2a-c), and 60° (3a-c). a) Mean TKE in LES- b) Relative travel of particles in Z-direction between two consecutive PIV images $Z_{travel,rel}$, estimated based on phase-averaged Z-velocity predictions of the LES. c) Phase-averaged PIV correlation factor of the PIV analysis, overlayed with the threshold isolines of the Mean TKE = 0.5 J/kg (black) and $Z_{travel,rel}$= 0.2 (white).

D. Comparison between LES & RANS

1. Phase averaged velocity fields

Fig. S9 and Fig. S10 show the comparison of phase-averaged velocity fields between LES and RANS as shown and discussed in the manuscript for additional main-blade angles of 0° and 60°, respectively.

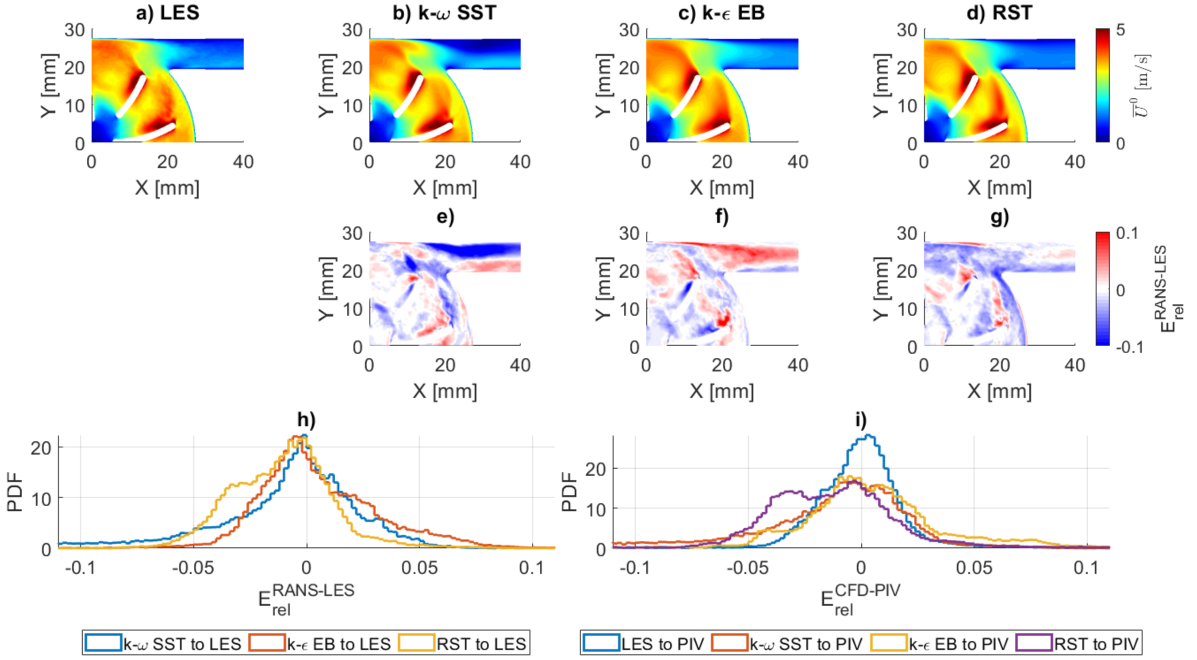

Fig. S9 *Velocity fields for the LES and RANS and their respective deviations for the impeller position at 0°. a) - d) Phase averaged velocity magnitude fields* $\overline{U}^0$ *of LES, k-ω SST, k-ε EB, RST (from left to right). e) - g) Relative deviation of the* $\overline{U}^0$ *predictions* $\left(E_{rel}(\overline{U}^0)\right)$ *between the RANS and the LES as defined in the manuscript. h) PDF of* $E_{rel}(\overline{U}^0)$ *between RANS and LES. i) PDF of* $E_{rel}(\overline{U}^0)$ *between CFD and PIV.*

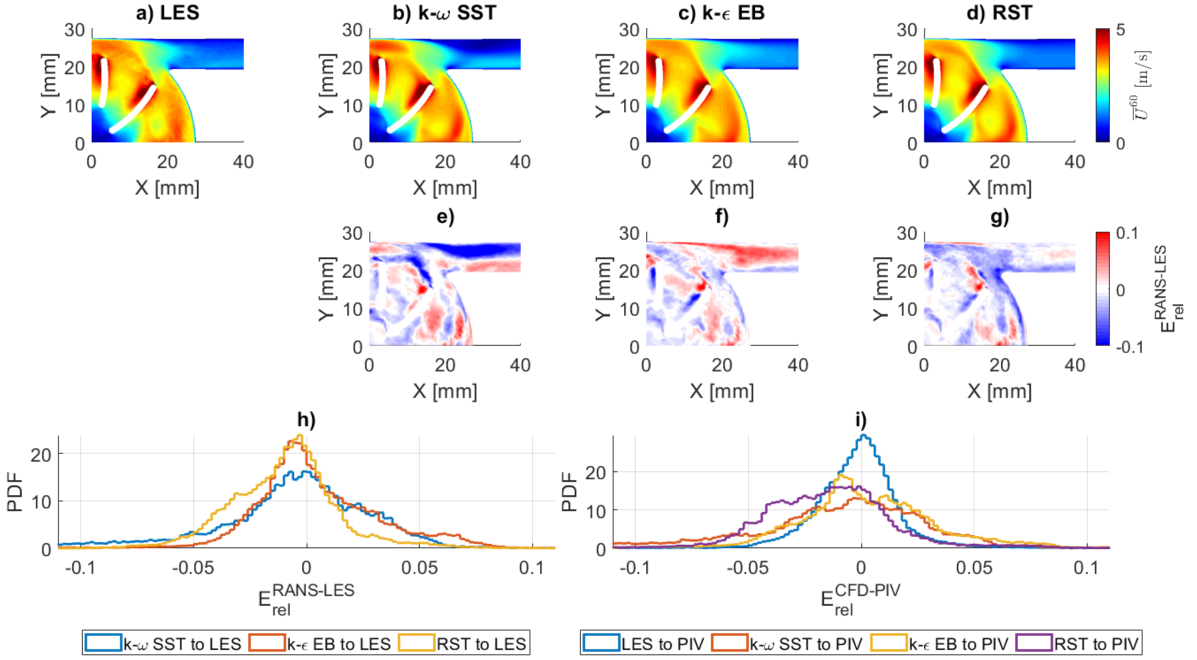

Fig. S10 *Velocity fields for the LES and RANS and their respective deviations for the impeller position at 60°. a) - d) Phase averaged velocity magnitude fields* $\overline{U}^{60}$ *of LES, k-ω SST, k-ε EB, RST (from left to right). e) - g) Relative deviation of the* $\overline{U}^{60}$ *predictions* $\left(E_{rel}(\overline{U}^{60})\right)$ *between the RANS and the LES as defined in the manuscript. h) PDF of* $E_{rel}(\overline{U}^{60})$ *between RANS and LES. i) PDF of* $E_{rel}(\overline{U}^{60})$ *between CFD and PIV.*

2. Phase Averaged Pressure Fields

Fig. S11, Fig. S12 and Fig. S13 show the comparison of the phase-averaged pressure fields between LES and RANS for phase angles of 0°, 30° and 60°, respectively. While the pressure boundary condition was set as 0 mmHg at the outlet for all simulations, the depicted pressure fields show the relative pressure to the inlet, as it allows a more intuitive interpretation. All impeller positions show similar results. The k-ω SST model shows the best overall performance, with a very good mean agreement with the LES and larger deviations mostly localized in the blade wake and the stagnation point at the volute exit. The k-ε EB model shows a mean deviation from the LES, caused by a general overprediction of pressure. The RST model is unique in showing two distinct peaks in its PDF, one around a deviation of 0 to 1 mmHg and a second around -4 mmHg. The second peak is caused by a failure to accurately predict the pressure increase from the volute into the outlet, leading to and underprediction of the pressure in the entire outlet cannula.

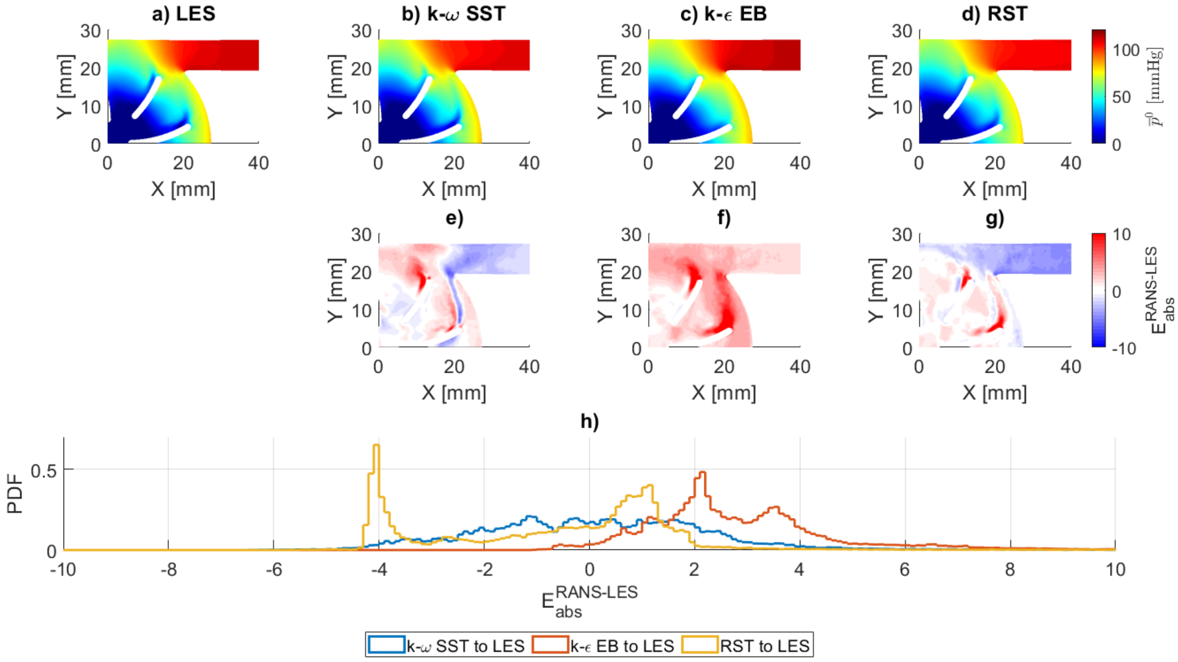

Fig. S11 *Pressure fields for the LES and RANS and their respective deviations for the impeller position at 0°. a) - d) Phase averaged pressure magnitude fields $\bar{p}^0$ of LES, k-ω SST, k-ε EB, RST (from left to right). e) - g) Absolute deviation of the $\bar{p}^0$ predictions $(E_{abs}(\bar{p}^0))$ between the RANS and the LES. h) PDF of $E_{abs}(\bar{p}^0)$ between RANS and LES.*

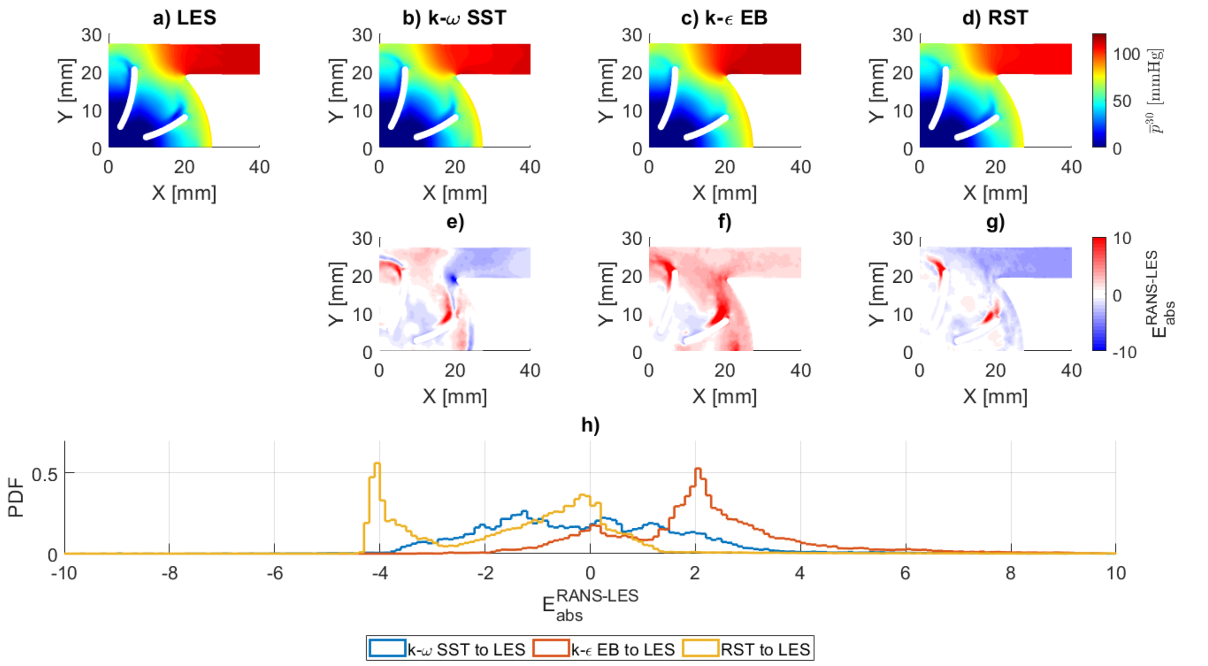

Fig. S12 *Pressure fields for the LES and RANS and their respective deviations for the impeller position at 30°. a) - d) Phase averaged pressure magnitude fields $\bar{p}^{30}$ of LES, k-ω SST, k-ε EB, RST (from left to right). e) - g) Absolute deviation of the $\bar{p}^{30}$ predictions $\left(E_{abs}(\bar{p}^{30})\right)$ between the RANS and the LES. h) PDF of $E_{abs}(\bar{p}^{30})$ between RANS and LES.*

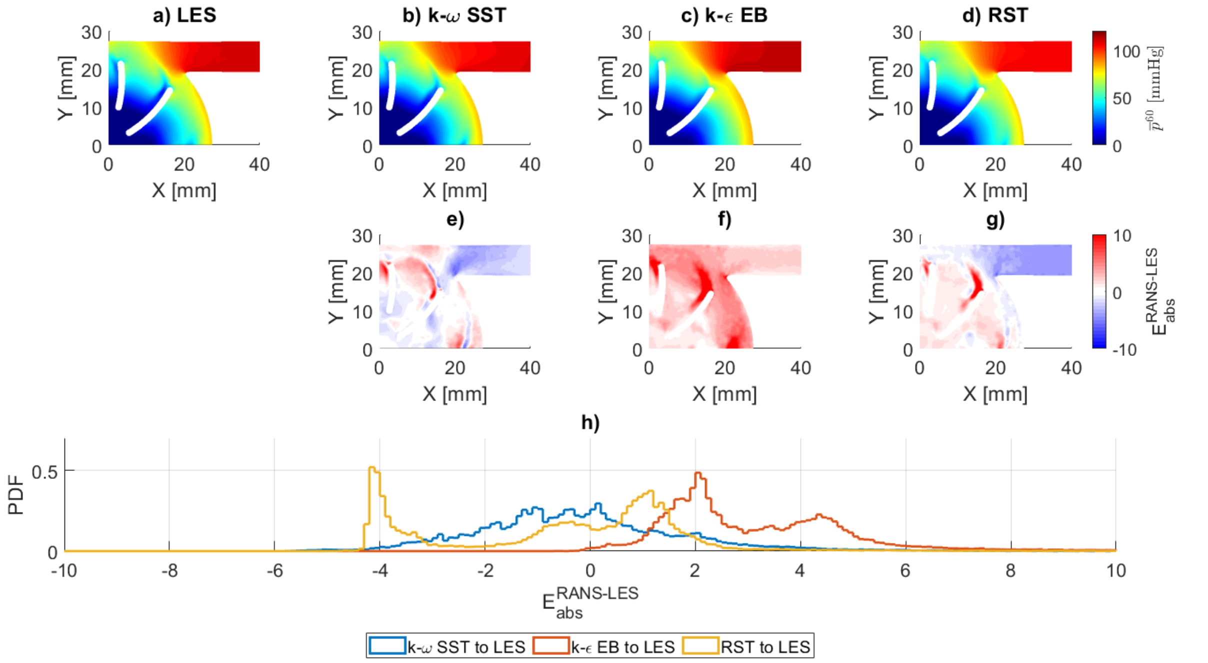

Fig. S13 *Pressure fields for the LES and RANS and their respective deviations for the impeller position at 60°. a) - d) Phase averaged pressure magnitude fields $\bar{p}^{60}$ of LES, k-ω SST, k-ε EB, RST (from left to right). e) - g) Absolute deviation of the $\bar{p}^{60}$ predictions $\left(E_{abs}(\bar{p}^{60})\right)$ between the RANS and the LES. h) PDF of $E_{abs}(\bar{p}^{60})$ between RANS and LES.*
